# Transmissive Terahertz Tunable and Multifunctional Polarization Manipulation With Ge/VO_2_ Hybrid Metasurface

**DOI:** 10.1002/nap2.70175

**Published:** 2026-06-19

**Authors:** Xingxing Fan, Qiang Bian, Qingguo Du, Song Sun, Qin Fu, Zhengying Li, Fei Xu

**Affiliations:** ^1^ School of Information Engineering Wuhan University of Technology Wuhan China; ^2^ School of Electrical Engineering Naval University of Engineering Wuhan China; ^3^ Microsystem and Terahertz Research Center China Academy of Engineering Physics Chengdu China; ^4^ Institute of Electronic Engineering China Academy of Engineering Physics Mianyang China; ^5^ School of Information Engineering National Engineering Research Center of Optical Fiber Sensing Technology and Networks Wuhan University of Technology Wuhan China; ^6^ National Laboratory of Solid State Microstructures College of Engineering and Applied Sciences Nanjing University Nanjing China

**Keywords:** asymmetric transmission, multifunctional polarization manipulation, spin‐dependent, tunable

## Abstract

To meet the growing demand for transmissive tunable multifunctional polarization manipulation devices, a tunable multifunctional polarization‐manipulating metasurface (TMPMM) based on germanium dielectric pillars and $VO_{2}$ grating is proposed. When the $VO_{2}$ grating is in the insulating state, efficient cross‐polarization conversion in the frequency range of 3.88–5.5 THz is achieved, with a maximum conversion efficiency exceeding 0.98. Within the frequency ranges of 2.31–2.61 THz and 6.42–6.68 THz, the linearly polarized (LP) wave is efficiently converted to a circularly polarized (CP) wave. When the $VO_{2}$ grating is in the metallic state, the function of asymmetric transmission (AT) is realized, where the TMPMM can be utilized as a broadband isolator and half‐wave plate for an x‐polarized wave and can also be utilized as a narrowband isolator and polarization conversion switch for a y‐polarized wave. Under the forward incidence of CP waves, the TMPMM selectively transmits left‐ and right‐circularly polarized waves near 2.25 THz and 6.37 THz, respectively, achieving significant spin‐dependent polarization selection. Additionally, through simple temperature tuning, the proposed TMPMM enables dynamic control of AT, circular dichroism, and ellipticity. All these multifunctional and tunable properties are realized via temperature variation without altering the structural geometry parameters.

## Graphical abstract

A hybrid metasurface composed of Ge dielectric pillars and a VO₂ grating is proposed, which enables functional switching between symmetric and asymmetric polarization conversion in transmission. By continuously tuning the conductivity of VO_2_, the structure achieves multiple dynamic controls of asymmetric transmission, circular dichroism, and ellipticity. Combined with metasurface rotation, broadband full‐Stokes polarization control can be further realized.

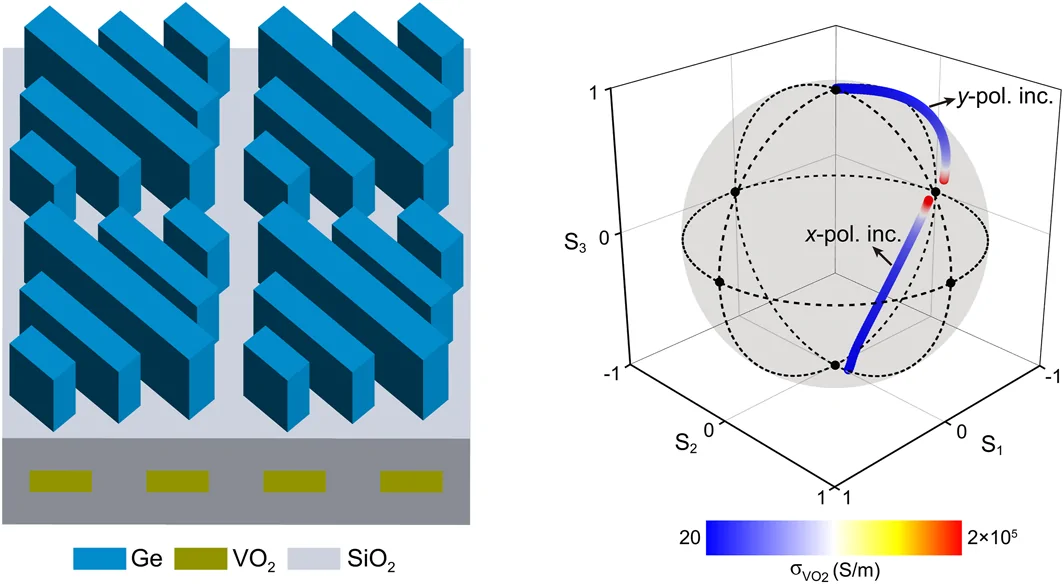

## 1 Introduction

Owing to the unique properties of good penetration, low photon energy, and rich spectral information, terahertz (THz) waves have been widely employed in many applications such as wireless communication, biomedical imaging, nondestructive testing, and material spectral analysis [1, 2, 3, 4, 5]. Flexible manipulation of the polarization state of a THz wave plays a crucial role for realizing the above‐mentioned applications. However, conventional polarization manipulation based on birefringence or the Faraday effect normally suffers from inherent limitations, including narrow operational bandwidth, bulky size, and high insertion losses, which severely hinder the development of polarization manipulation devices toward miniaturization, integration, and high performance [6, 7]. To overcome these limitations, metasurface‐based polarization manipulation devices have attracted attention from researchers, which can enable efficient control of polarization states by designing anisotropic meta‐atoms with enhanced light–matter interactions [8, 9, 10, 11].

To date, polarization manipulation devices based on metasurfaces with different materials or structures have been widely studied [12, 13, 14]. Among these, the metal‐insulator‐metal (MIM) configuration serves as a common one [15, 16, 17]. By carefully engineering the thickness of the dielectric layer to compensate for the intrinsic dispersion generated by the resonance of the metal structure, broadband and dispersion‐free polarization conversion can be achieved in reflection mode [18]. However, reflective metasurfaces suffer from inherent issues such as feed blockage and reliance on additional beam‐steering components, making them difficult to integrate into optical paths as conveniently as transmissive devices [19, 20, 21, 22]. Therefore, the development of transmissive polarization‐control metasurfaces that combine high performance with multifunctionality holds significant application prospects [23]. Because of the inherently high reflectivity of metallic materials in the THz wave [24], polarization converters composed solely of metallic resonators are hard to achieve high conversion efficiency with transmission efficiencies exceeding 0.9 over a broad wavelength range [25, 26, 27]. This limitation severely restricts the polarization conversion performance and working bandwidth of optoelectronic devices when applied in imaging and communication applications [28, 29]. To improve the transmission efficiency, researchers have proposed structures integrating orthogonal metal gratings on the top and bottom of metallic resonators [30, 31, 32, 33]. These designs leverage Fabry–Perot‐like cavity resonances to increase cross‐polarization conversion efficiency [34]. However, this approach relies primarily on the polarization‐selective properties of the front and back gratings, limiting polarization control to linearly polarized (LP) waves. To overcome the limitation and enable efficient manipulation of multi‐polarization states, dielectric metasurfaces composed of structures with a high aspect ratio and materials with a high refractive index have emerged as promising alternatives [35, 36, 37]. Dielectric metasurfaces can excite displacement current resonances, induce strong internal reflection at the interface between the nanostructures and the surrounding low‐refractive‐index media, and effectively trap energy in the form of standing waves while avoiding ohmic losses, thereby establishing the physical foundation for efficient multi‐polarization‐state control [38]. Recently, dielectric metasurfaces have been successfully applied to various functional polarization devices such as half‐wave plates (HWPs) [39], quarter‐wave plates (QWPs) [40], polarization splitters [41], and polarization switches [42]. However, with fixed geometric parameters, most dielectric metasurfaces can achieve only a single function and lack dynamic‐control capabilities. Consequently, enabling a dielectric metasurface to possess high‐efficiency tunable and multifunctional polarization manipulation without altering the unit structure remains a challenge in this research field.

In this work, we propose a terahertz tunable multifunctional polarization‐manipulating metasurface (TMPMM), which consists of cascaded germanium (Ge) dielectric pillars and $VO_{2}$ grating. The designed metasurface can support multiple functional operations, including HWP, QWP, asymmetric transmission (AT), polarization conversion switching, and spin‐dependent polarization selectivity. Specifically, at 300 K, $VO_{2}$ is in the insulating state, and the proposed metasurface efficiently converts an LP wave into its orthogonal counterpart over a broadband range of 3.88 THz–5.5 THz, with transmittances exceeding 0.98 near 4.8 THz and a polarization conversion ratio (PCR) reaching 0.999, demonstrating a near‐perfect function as HWP. Within dual frequency bands of 2.31 THz–2.61 THz and 6.42 THz–6.68 THz, the proposed metasurface efficiently transforms an LP wave into a circularly polarized (CP) wave, with an ellipticity above 0.9, realizing a high‐performance QWP. When the temperature is raised to 340 K, $VO_{2}$ is in the metallic state, and the proposed metasurface shows a significant AT response across 3.68 THz–5.87 THz (AT>0.8), with a maximum AT of 0.958, making it highly suitable for optical isolators and polarization switches. Under CP forward illumination, the structure selectively transmits a left‐circularly polarized (LCP) wave near 2.25 THz and a right‐circularly polarized (RCP) wave near 6.37 THz, exhibiting circular dichroism (CD) values as high as 0.917 and −0.83, respectively. These results indicate that the structure exhibits significant spin‐dependent polarization selectivity, offering new insights into constructing robust CP/LP wave generators. Through Jones matrix analysis and multipole decomposition, the underlying physical mechanisms of polarization manipulation are systematically discussed. Furthermore, with only temperature tuning, the metasurface enables dynamic multidimensional control over AT, CD, and ellipticity, including flexible switching between symmetric and asymmetric transmission, transition from nonchiral to strongly chiral responses, and continuous modulation between CP and LP waves. The proposed TMPMM not only integrates diverse polarization‐manipulation capabilities but also enables dynamic reconfiguration, paving the way for further exploration in polarization optics and chiral photonics.

## 2 Structure Design and Methods

The schematic diagram of the proposed TMPMM is illustrated in Figure 1. Each unit cell comprises a bilayer structure, with the upper layer consisting of five Ge dielectric pillars placed on a $SiO_{2}$ substrate with an $VO_{2}$ grating embedded in the substrate. In the terahertz regime, the refractive index of Ge is 3.96 with low loss [43]. The refractive index of the $SiO_{2}$ substrate is 1.45 [44]. The relative permittivity of $VO_{2}$ can be described by the Drude model [6].

(1) $$\varepsilon \left(\omega \right)=\varepsilon_{\infty }-\frac{\omega_{p}^{2}\left(\sigma_{VO_{2}}\right)}{\omega^{2}+i\gamma \omega }$$

in which $\varepsilon_{\infty }=12$ represents the relative permittivity of $VO_{2}$ at infinite frequency, $\gamma =5.75\times 10^{13}$ rad/s denotes the collision frequency, and $\omega_{p}\left(\sigma_{VO_{2}}\right)$ denotes the plasma frequency, which varies with conductivity according to $\omega_{p}^{2}\left(\sigma_{VO_{2}}\right)=\sigma_{VO_{2}}\omega_{p}^{2}\left(\sigma_{0}\right)/\sigma_{0}$, where $\sigma_{0}=3\times 10^{5}$ S/m and $\omega_{p}\left(\sigma_{0}\right)=1.4\times 10^{15}$ rad/s. Figure 1b shows a top view of the top layer of the unit cell in the x‐y plane, where all Ge dielectric pillars are aligned along the u‐axis at an angle of ξ=45° relative to the x‐axis. The top layer remains in‐plane $C_{2v}$ symmetry, enabling efficient conversion of an incident LP wave into its orthogonally polarized wave or a CP wave. Figure 1c presents a top view of the bottom layer within one unit, where the $VO_{2}$ grating is aligned with the x‐axis. Dynamic polarization manipulation is achieved by switching the conductivity state of $VO_{2}$ (insulating or metallic state) through temperature modulation. Unlike previously reported front‐back bilayer grating configurations [30, 31, 32, 33, 34], here, only one layer of grating is employed, which not only offers a simpler design but also enables modulation of the CP wave, providing an additional degree of freedom that enhances flexibility for spin‐dependent manipulation. The modeling and simulation in this work are performed using the finite element method [7]. Perfectly matched layers are applied as absorbing boundary conditions in the *z*‐direction, whereas periodic boundary conditions are employed along the x‐ and y‐directions.

**FIGURE 1:**
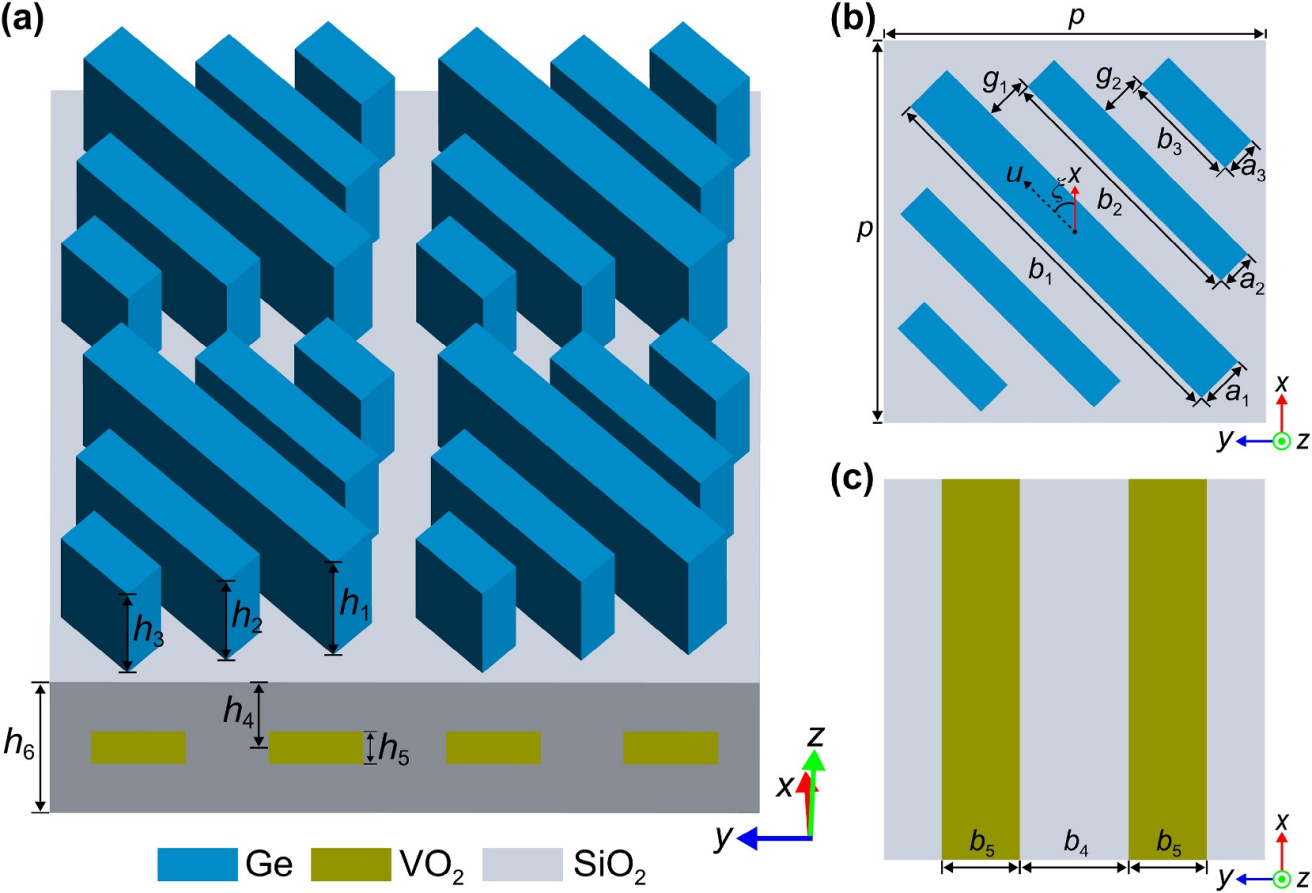
Schematic of the proposed tunable multifunctional polarization‐manipulating metasurface (TMPMM). (a) Four unit cells, with each unit cell consisting of five Ge dielectric pillars placed on top of a homogeneous ${SiO}_{2}$ substrate with embedded ${VO}_{2}$ gratings. Top view of (b) the top layer and (c) the substrate in the x‐y plane. Geometric parameters are set as $h_{1}=49$ μm, $h_{2}=h_{3}=40$ μm, $h_{4}=15$ μm, $h_{5}=1.467$ μm, $h_{6}=30$ μm, p=12 μm, $a_{1}=1$ μm, $a_{2}=a_{3}=0.8$ μm, $b_{1}=14$ μm, $b_{2}=10$ μm, $b_{3}=5$ μm, $g_{1}=1.6$ μm, $g_{2}=1.7$ μm, ξ=45°, and $b_{4}=b_{5}=2.88$ μm.

Considering a normally incident plane wave propagating along the z‐axis, as shown in Figure 1, the transmission properties can be described by the following Jones matrix:

(2a) $$\left(\begin{array}{c} E_{x}^{t}\\ E_{y}^{t} \end{array}\right)=A\left(-\xi \right)\left[\begin{array}{cc} t_{uu} & t_{uv}\\ t_{vu} & t_{vv} \end{array}\right]A\left(\xi \right)\left(\begin{array}{c} E_{x}^{i}\\ E_{y}^{i} \end{array}\right)=\left[\begin{array}{cc} t_{xx} & t_{xy}\\ t_{yx} & t_{yy} \end{array}\right]\left(\begin{array}{c} E_{x}^{i}\\ E_{y}^{i} \end{array}\right)$$

where A(ξ) denotes the rotation matrix, defined as follows:

(2b) $$\left[\begin{array}{cc} cos\left(\xi \right) & sin\left(\xi \right)\\ -sin\left(\xi \right) & cos\left(\xi \right) \end{array}\right]$$

Substituting ξ=45° into Equation (2a) yields the following equations:

(3a) $$t_{xx}=\left[\left(t_{uu}-t_{uv}\right)-\left(t_{vu}-t_{vv}\right)\right]/2$$

(3b) $$t_{xy}=\left[\left(t_{uu}+t_{uv}\right)-\left(t_{vu}+t_{vv}\right)\right]/2$$

(3c) $$t_{yx}=\left[\left(t_{uu}-t_{uv}\right)+\left(t_{vu}-t_{vv}\right)\right]/2$$

(3d) $$t_{yy}=\left[\left(t_{uu}+t_{uv}\right)+\left(t_{vu}+t_{vv}\right)\right]/2$$

where $t_{uu}=a_{uu}e^{i\Phi_{uu}}$ and $t_{vv}=a_{vv}e^{i\Phi_{vv}}$ represent the co‐polarized transmission coefficients for u‐ and v‐polarized incident waves, respectively, whereas $t_{vu}=a_{vu}e^{i\Phi_{vu}}$ and $t_{uv}=a_{uv}e^{i\Phi_{uv}}$ represent the corresponding cross‐polarized coefficients. Here, u and v represent the long‐axis and short‐axis directions of the TMPMM, respectively, as shown in Figure 1b. Similarly, $t_{xx}$, $t_{yy}$, and $t_{yx}$, $t_{xy}$ correspond to the co‐polarized and cross‐polarized transmission coefficients for x‐ and y‐polarized incidence, respectively. The superscripts t and i are used to indicate transmitted and incident waves. For the transmission characteristics of CP waves, the coefficients can be expressed in terms of the LP coefficients defined in Equation (2a) as follows [6]:

(4a) $$t_{++}=\left[t_{xx}+t_{yy}+i\left(t_{xy}-t_{yx}\right)\right]/2$$

(4b) $$t_{+-}=\left[t_{xx}-t_{yy}-i\left(t_{xy}+t_{yx}\right)\right]/2$$

(4c) $$t_{-+}=\left[t_{xx}-t_{yy}+i\left(t_{xy}+t_{yx}\right)\right]/2$$

(4d) $$t_{--}=\left[t_{xx}+t_{yy}-i\left(t_{xy}-t_{yx}\right)\right]/2$$

where +/− represents an RCP/LCP wave, and an RCP/LCP wave is defined as the electric vector rotating clockwise/counterclockwise, respectively, when the observer looks in the opposite direction of wave propagation.

To evaluate the performance of the TMPMM as a multifunctional polarization manipulation device, three key metrics, normalized ellipticity η, PCR, and AT parameter Δ are introduced. Generally, η characterizes the polarization state of the THz wave, PCR quantitatively describes the efficiency with which the TMPMM converts the polarization state of an incident wave to its orthogonal state, and Δ indicates the transmission difference between forward‐ and backward‐propagating waves. These parameters are defined as follows [32, 45]:

(5) $$\eta =\frac{1}{2}\sin^{-1}\left(\frac{S_{3}}{S_{0}}\right)/45$$

(6a) $${PCR}_{y}^{f/b}={\left|t_{yx}^{f/b}\right|}^{2}/\left({\left|t_{xx}^{f/b}\right|}^{2}+{\left|t_{yx}^{f/b}\right|}^{2}\right)=T_{yx}^{f/b}/\left(T_{xx}^{f/b}+T_{yx}^{f/b}\right)$$

(6b) $${PCR}_{x}^{f/b}={\left|t_{xy}^{f/b}\right|}^{2}/\left({\left|t_{yy}^{f/b}\right|}^{2}+{\left|t_{xy}^{f/b}\right|}^{2}\right)=T_{xy}^{f/b}/\left(T_{yy}^{f/b}+T_{xy}^{f/b}\right)$$

(7a) $$\Delta_{\text{lin}}^{x}={\left|t_{xx}^{f}\right|}^{2}+{\left|t_{yx}^{f}\right|}^{2}-{\left|t_{xx}^{b}\right|}^{2}-{\left|t_{yx}^{b}\right|}^{2}=T_{xx}^{f}+T_{yx}^{f}-T_{xx}^{b}-T_{yx}^{b}$$

(7b) $$\Delta_{\text{lin}}^{y}={\left|t_{yy}^{f}\right|}^{2}+{\left|t_{xy}^{f}\right|}^{2}-{\left|t_{yy}^{b}\right|}^{2}-{\left|t_{xy}^{b}\right|}^{2}=T_{yy}^{f}+T_{xy}^{f}-T_{yy}^{b}-T_{xy}^{b}$$

Here, $S_{0}$ and $S_{3}$ denote Stokes parameters [46]; the superscripts f and b represent the directions of forward (along the −z direction) and backward (along the +z direction) propagation, respectively. $T_{xx}={\left|t_{xx}\right|}^{2}$, $T_{yy}={\left|t_{yy}\right|}^{2}$, $T_{yx}={\left|t_{yx}\right|}^{2}$, and $T_{xy}={\left|t_{xy}\right|}^{2}$ denote the co‐ and cross‐polarized transmittances for x‐ and y‐polarized incident waves, respectively.

## 3 Results and Discussion

### 3.1 Multifunctional Polarization Converter

At room temperature (300 K), $VO_{2}$ is in the insulating state with $\sigma_{{\text{VO}}_{2}}=20$ S/m [6]. Figure 2a,b shows the simulated co‐ and cross‐polarized transmission spectra of the TMPMM under forward and backward incidence of LP waves, respectively. The results indicate that under both forward and backward excitation, the TMPMM can efficiently convert x‐ and y‐polarized waves into waves with orthogonal polarization states over a broad frequency band. One may notice that there are slight differences in the co‐ and cross‐polarized transmittances. This mainly originates from the different modulation effects exerted by the high‐contrast $VO_{2}$ grating on x‐ and y‐polarized waves [47]; see Supporting Information S1: Figure S1a in Note S1. For forward x‐ and y‐polarized incident waves, the cross‐polarized transmittances exceed 0.8 in the ranges of 3.58 THz–5.77 THz and 3.6 THz–5.66 THz, respectively, reaching maxima of 0.988 at 4.8 THz and 0.98 at 4.9 THz, which demonstrate that the proposed metasurface is a near‐perfect HWP in the working frequency bands. For the HWP function under CP waves, incidents are provided in Supporting Information S1: Note S2. When the x‐ and y‐polarized waves are incident from the backward direction, they are first modulated by the $VO_{2}$ grating and subsequently converted into the orthogonal polarization state by the Ge dielectric pillars. Consequently, the cross‐polarized transmittances for backward‐incident x‐ and y‐polarized waves are interchanged compared to those of the forward case, whereas the co‐polarized transmittance remains the same. As a result, the co‐ and cross‐polarized curves intersect at different points for different incident polarizations or different propagation directions, as indicated by the blue and red arrows in Figure 2a,b, where the insets show enlarged views of the gray‐shaded regions. Because CP waves can be regarded as the coherent superposition of x‐ and y‐polarized waves, this asymmetric response further leads to polarization‐ and direction‐dependent conversion from LP to CP waves, as shown in Figure 2c,d. In the low‐frequency region around 2.45 THz, forward‐incident x‐ and y‐polarized waves are efficiently converted into LCP and RCP waves, respectively, with maximum conversion efficiencies reaching 0.928 and 0.927. Simultaneously, the corresponding orthogonal components are both below 0.005, realizing near‐perfect QWP performance. For backward incidence, the x‐ and y‐polarized waves are efficiently converted to RCP and LCP waves, respectively, with the chirality inversion of CP waves originating from the change in the observation direction [48]. The resulting RCP and LCP waves exhibit maximum transmittances of 0.917 and 0.937, respectively, with orthogonal components as low as 0.0031 and 0.0037, also exhibiting a near‐perfect function as a QWP. In the high‐frequency region around 6.55 THz, forward‐incident x‐ and y‐polarized waves can still be efficiently converted into RCP and LCP waves, but the corresponding orthogonal components are relatively larger (minimum values of 0.0147 and 0.062, respectively), which limits the ellipticity of the transmitted waves. In contrast, for backward‐incident x‐ and y‐polarized waves, high‐efficiency CP conversion is achieved, while the orthogonal components are effectively suppressed below 0.005, with minima reaching $4\times 10^{-4}$ and $7\times 10^{-4}$, respectively. Therefore, in the low‐frequency region, the proposed TMPMM achieves near‐perfect CP conversion for both forward‐incident and backward‐incident LP waves. In the high‐frequency region, to attain high‐quality CP conversion, backward illumination of the TMPMM is preferred.

**FIGURE 2:**
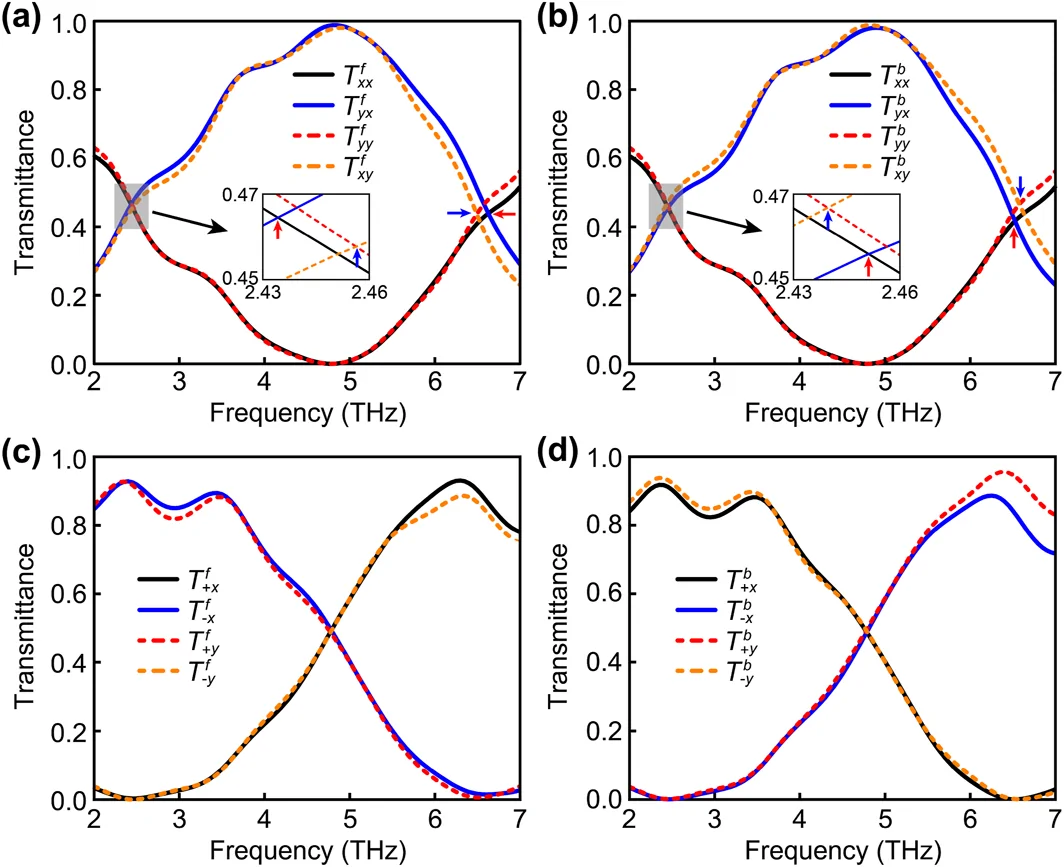
Transmission spectra of co‐ and cross‐polarized waves for (a) forward‐ and (b) backward‐incident x‐ and y‐polarized waves at room temperature, respectively. The insets show enlarged views of the gray shaded area. Transmission spectra for (c) forward‐ and (d) backward‐incident RCP and LCP waves, respectively.

Furthermore, based on Equations ((5), (6a), (6b), (7a), (7b)), the calculated PCR, η, and AT are systematically analyzed to evaluate the performance of the TMPMM, with results shown in Figure 3a–c. As shown in Figure 3a, the PCRs differ only slightly between forward‐ and backward‐incident x‐ and y‐polarized waves. Particularly, at high conversion efficiencies, the four curves nearly overlap with each other, as illustrated in the inset of Figure 3a. Thus, across the broad frequency range of 3.88 THz–5.5 THz, the PCRs for both x‐ and y‐polarized waves exceed 0.9 regardless of the incident direction. This demonstrates that the TMPMM can simultaneously achieve broadband cross‐polarization conversion for both polarization states under different illumination directions. Furthermore, at 4.8 THz, the PCRs for both forward‐ and backward‐incident x‐ and y‐polarized waves exceed 0.999; the corresponding ellipticities are close to 0, as shown in Figure 3b. This indicates that the incident LP waves are ideally converted into orthogonally polarized waves. Consequently, the TMPMM exhibits a high‐performance HWP. Additionally, due to the small difference in orthogonal polarization conversion between the two incident directions, the structure remains transmission symmetric (i.e., AT≈0) across the broadband around 4.8 THz, as indicated by the gray‐shaded area in Figure 3c. Regarding polarization conversion from LP to CP waves, η in Figure 3b reveals that the conversion occurs near 2.45 THz and 6.55 THz. Specifically, in the low‐frequency region, the forward‐incident ellipticities of $-\eta_{x}^{f}$ and $\eta_{y}^{f}$ exceed 0.9 within the ranges of 2.34 THz–2.58 THz and 2.3 THz–2.62 THz, respectively, reaching maxima of 0.925 at 2.45 THz and 0.997 at 2.46 THz. For backward incidence, $\eta_{x}^{b}$ and $-\eta_{y}^{b}$ exceed 0.9 within the ranges of 2.3 THz–2.64 THz and 2.31 THz–2.61 THz, respectively, with maxima of 0.97 at 2.47 THz and 0.956 at 2.45 THz. This implies that both forward‐ and backward‐incident LP waves are effectively converted to CP waves, confirming the bidirectional function as QWP of the TMPMM in the low‐frequency region. In the high‐frequency region, the forward‐incident ellipticities $\eta_{x}^{f}$ and $-\eta_{y}^{f}$ reach maxima of 0.836 at 6.59 THz and 0.893 at 6.51 THz, respectively, whereas the ellipticities of backward‐incident waves, $-\eta_{x}^{b}$ and $\eta_{y}^{b}$, exceed 0.9 within the ranges of 6.37 THz–6.68 THz and 6.42 THz–6.75 THz, respectively, with maxima of 0.975 at 6.52 THz and 0.966 at 6.57 THz. Hence, in the high‐frequency region, the TMPMM with backward incidence can be adopted to effectively achieve broadband CP conversion for both x‐ and y‐polarized incident waves. In summary, at room temperature, the proposed TMPMM simultaneously achieves the functionalities of high‐efficiency broadband HWP and QWP.

**FIGURE 3:**
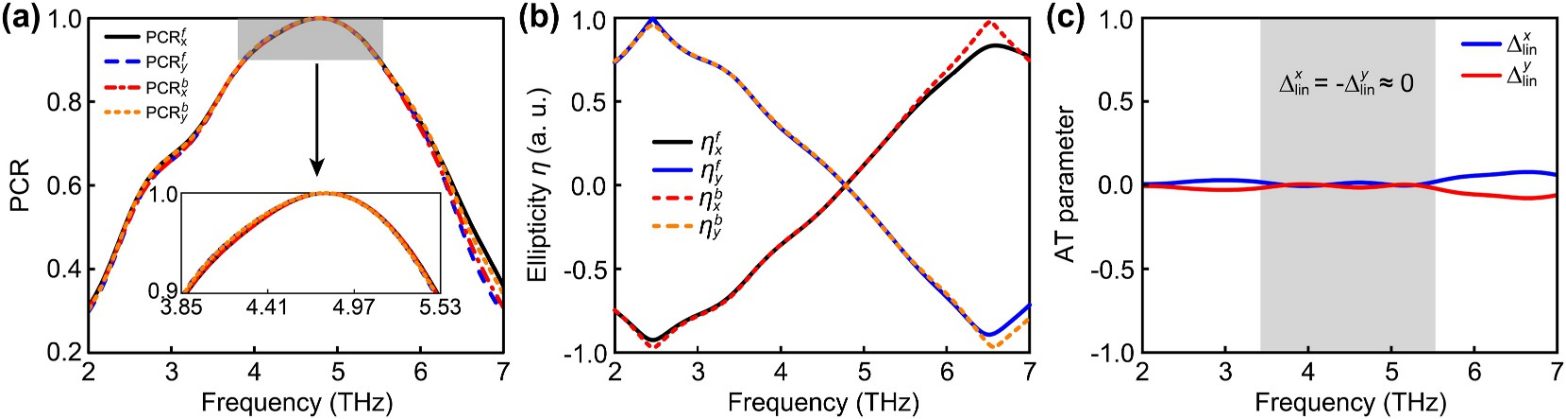
(a) PCR and (b) ellipticity curves for forward‐ and backward‐incident x‐ and y‐polarized waves. (c) AT parameters.

To better understand the polarization conversion mechanisms of the TMPMM at room temperature, a detailed analysis is conducted using the Jones matrix, taking the forward‐incident x‐polarized wave as an example. The electric field of the incident wave can be expressed by the Jones vector as ${\left[\begin{array}{cc} 1 & 0 \end{array}\right]}^{T}$. Substituting this into Equation (2a) yields the mathematical expression for the transmitted electric field:

$$\left(\begin{array}{c} E_{x}^{t\left(f\right)}\\ E_{y}^{t\left(f\right)} \end{array}\right)=\left[\begin{array}{c} t_{xx}^{f}\\ t_{yx}^{f} \end{array}\right]=\frac{1}{2}\left[\begin{array}{c} \left(t_{uu}-t_{uv}\right)-\left(t_{vu}-t_{vv}\right)\\ \left(t_{uu}-t_{uv}\right)+\left(t_{vu}-t_{vv}\right) \end{array}\right]$$

(8) $$=\frac{1}{2}e^{i\Phi_{vv}}\left[\begin{array}{c} a_{uu}e^{i\Delta \Phi_{1}}+a_{vv}-a_{vu}e^{i\Delta \Phi_{2}}-a_{uv}e^{i\Delta \Phi_{3}}\\ a_{uu}e^{i\Delta \Phi_{1}}+a_{vu}e^{i\Delta \Phi_{2}}-a_{uv}e^{i\Delta \Phi_{3}}-a_{vv} \end{array}\right]$$

Here, $\Delta \Phi_{1}=\Phi_{uu}-\Phi_{vv}$, $\Delta \Phi_{2}=\Phi_{vu}-\Phi_{vv}$, and $\Delta \Phi_{3}=\Phi_{uv}-\Phi_{vv}$. Figure 4a,b shows the amplitude and phase difference distributions of the transmission coefficients with u‐ and v‐polarized incidence at room temperature, respectively.

**FIGURE 4:**
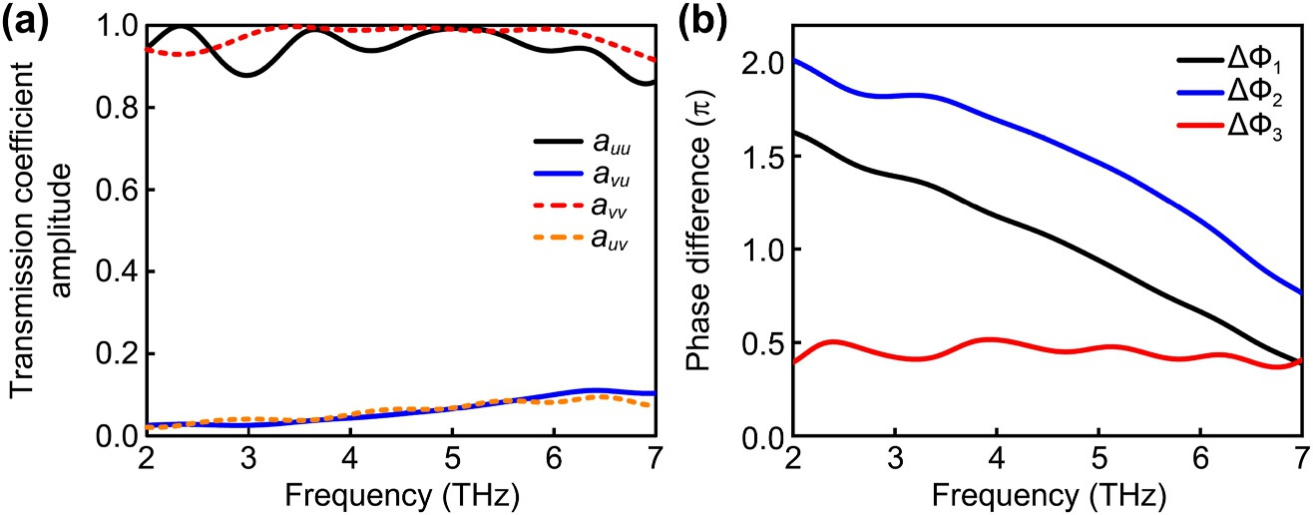
(a) Co‐polarized (black solid and red dashed) and cross‐polarized (blue solid and orange dashed) transmission coefficients with u‐ and v‐polarized incidence at room temperature. (b) Phase differences between $t_{uu}$ and $t_{vv}$ (black curve), $t_{vu}$ and $t_{vv}$ (blue curve), and $t_{uv}$ and $t_{vv}$ (red curve).

At the frequency near 4.8 THz, due to $\Delta \Phi_{2}\approx 1.5\pi$, $\Delta \Phi_{3}\approx 0.5\pi$, and $a_{vu}\approx a_{uv}$, the terms $-a_{vu}e^{i\Phi_{2}}-a_{uv}e^{i\Phi_{3}}$ in $t_{xx}^{f}$ almost cancel each other. At this point, the transmitted electric field can be approximated as follows:

(9) $$\left(\begin{array}{c} E_{x}^{t\left(f\right)}\\ E_{y}^{t\left(f\right)} \end{array}\right)=\frac{1}{2}e^{i\Phi_{vv}}\left[\begin{array}{c} a_{uu}e^{i\Delta \Phi_{1}}+a_{vv}\\ a_{uu}e^{i\Delta \Phi_{1}}-2a_{uv}e^{i\Delta \Phi_{3}}-a_{vv} \end{array}\right]$$

In this frequency range, $a_{uu}$ and $a_{vv}$ remain close to 1, and $\Delta \Phi_{1}\approx \pi$. Hence, $|t_{xx}^{f}|\approx 0$, whereas $|t_{yx}^{f}|\approx 1$, achieving near‐perfect orthogonal polarization conversion.

In the low‐frequency region around 2.45 THz, both $a_{vu}$ and $a_{uv}$ are below 0.035 and are negligible compared with $a_{uu}$ and $a_{vv}$. The transmitted fields are simplified to the following:

(10) $$\left(\begin{array}{c} E_{x}^{t\left(f\right)}\\ E_{y}^{t\left(f\right)} \end{array}\right)=\frac{1}{2}e^{i\Phi_{vv}}\left[\begin{array}{c} a_{uu}e^{i\Delta \Phi_{1}}+a_{vv}\\ a_{uu}e^{i\Delta \Phi_{1}}-a_{vv} \end{array}\right]$$

Because of $a_{uu}$ and $a_{vv}$ being both high and comparable with each other in magnitude and $\Delta \Phi_{1}\approx 1.5\pi$, the LP wave is effectively converted to an LCP wave. In the high‐frequency region at 6.55 THz, $a_{vu}$ and $a_{uv}$ increase to 0.109 and 0.094, respectively, and their impact on polarization conversion must be considered. Substituting $a_{uu}=0.911$, $a_{vv}=0.957$, $\Delta \Phi_{1}=0.506\pi$, $\Delta \Phi_{2}=0.929\pi$, and $\Delta \Phi_{3}=0.382\pi$ into Equation (8) gives the following equation:

(11) $$\left(\begin{array}{c} E_{x}^{t\left(f\right)}\\ E_{y}^{t\left(f\right)} \end{array}\right)=\frac{1}{2}e^{i\Phi_{vv}+0.213\pi }\left[\begin{array}{c} 1.29\\ 1.4e^{i0.58\pi } \end{array}\right]$$

It can be seen that the amplitudes of $E_{x}^{t\left(f\right)}$ and $E_{y}^{t\left(f\right)}$ are slightly different, and their phase difference is larger than π/2, which limits the effect for conversion from LP to RCP waves. For forward y‐polarized incidence, a similar analysis can be performed by substituting ${\left[\begin{array}{cc} 0 & 1 \end{array}\right]}^{T}$ into Equation (2a), which is not repeated here. A detailed analysis of backward‐incident x‐polarized waves is provided in Supporting Information S1: Note S3.

The underlying physical mechanisms of polarization conversion can be further verified through multipole decomposition and field‐distribution analysis. The contributions of the electric dipole (ED), magnetic dipole (MD), electric toroidal dipole (ETD), magnetic toroidal dipole (MTD), electric quadrupole (EQ), and magnetic quadrupole (MQ) to far‐field radiation are calculated based on the induced current density [49]. Figure 5a,d shows the multipole decomposition results for forward‐incident u‐ and v‐polarized waves, respectively. Scattering power analysis indicates that, at 4.8 THz, where high‐efficiency orthogonal polarization conversion is achieved, the response for a u‐polarized incident wave is primarily governed by the MTD mode, whereas the v‐polarized incidence is jointly controlled by the MTD and ETD modes. The corresponding electric and magnetic field distributions on the right verify these observations, where the red arrows and white arrows represent current vectors and magnetic vectors, respectively. For u‐polarized incidence, the electric field is primarily localized around the dielectric pillars, forming three current loops on the u‐z plane with alternating rotational directions, as indicated by the red circles in Figure 5b. This indicates that three MD modes are excited along the v‐axis. Further analysis of the magnetic field distribution in Figure 5c shows that these three MD modes couple with each other to generate two counter‐rotating magnetic loops and excite the ${MTD}_{1}$ mode along the v‐axis, as illustrated schematically in Figure 5g. For v‐polarized incidence, the electric field similarly localizes around the dielectric pillars, forming two current loops with opposite rotations in the v‐z plane, marked by red circles in Figure 5e. This indicates that two opposing MD modes are excited along the v‐axis, thereby forming the ${ETD}_{2}$ mode along the v‐axis. Furthermore, the magnetic field distribution in Figure 5f reveals two counter‐rotating magnetic loops on the u‐z plane, exciting an ${MTD}_{3}$ mode along the u‐axis. Schematic diagrams of the ${ETD}_{2}$ and ${MTD}_{3}$ modes are shown in Figure 5h. Consequently, the transmission amplitude and phase with u‐polarized incidence are mainly modulated by the $MTD_{1}$ mode, whereas those with v‐polarized incidence are jointly regulated by the ${ETD}_{2}$ and ${MTD}_{3}$ modes, as depicted in Figure 5i. Because the field intensity excited by v‐polarized waves in the TMPMM is significantly lower than that for u‐polarized waves, the corresponding variations in amplitude and phase are relatively weaker, as shown in Figure 4. At a frequency of 4.8 THz, the modulated amplitudes of the u‐ and v‐polarized waves tend to be similar and approach unity, with a phase difference of approximately 180°, thereby enabling nearly perfect orthogonal polarization conversion.

**FIGURE 5:**
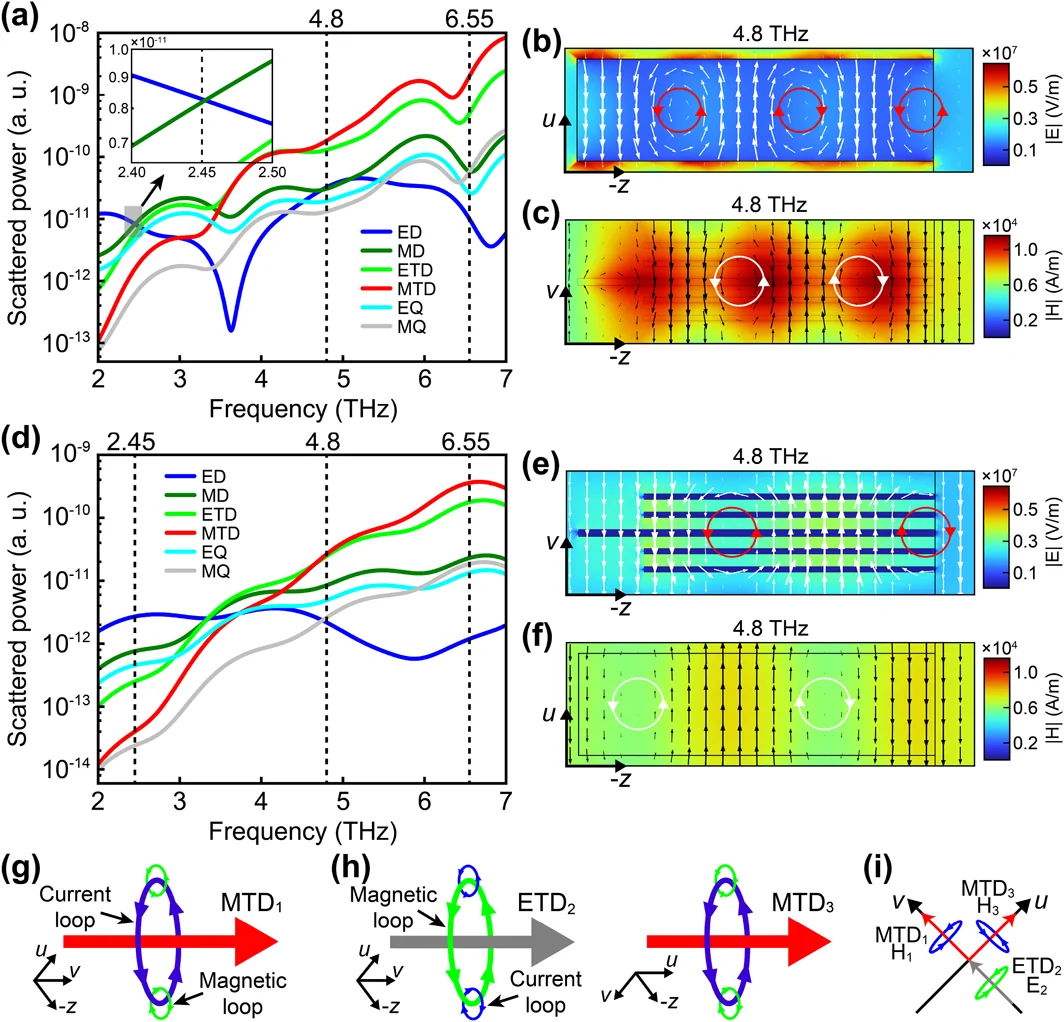
Mode analysis of polarization conversion. (a) Contributions of different multipole excitations with u‐polarized incidence. (b) Electric field distributions on the u‐z plane and (c) magnetic field distribution on the v‐z plane at 4.8 THz. (d) Contributions of different multipole excitations with v‐polarized incidence. (e) Electric field distribution on the v‐z plane and (f) magnetic field distribution on the u‐z plane at 4.8 THz. (g) and (h) Schematic illustrations of MTD and ETD formation. (i) ${MTD}_{1}$, ${ETD}_{2}$, and ${MTD}_{3}$. The color bars represent field magnitude. White and black arrows represent current vectors and magnetic vectors, respectively.

The conversion from LP to CP can also be interpreted through scattering power analysis. At 2.45 THz, with u‐polarized incidence, the response is dominated by the ${MD}_{1}$ and ${ED}_{2}$ modes [see inset in Figure 5a], whereas with v‐polarized incidence, the response mainly excites the ${ED}_{3}$ mode. At 6.55 THz, the modulation for both u‐ and v‐polarized waves is mainly controlled by MTD modes. Owing to the notable difference in field strengths excited by the two orthogonal polarizations, the modulated u‐ and v‐polarized waves exhibit distinct amplitudes and phases. When their amplitudes become comparable and the phase difference approaches 90°, effective conversion from LP to CP waves is achieved. Detailed EM field distributions and mode analyses are presented in Supporting Information S1: Note S4.

### 3.2 Broadband Asymmetric Transmission and Polarization Conversion Switching

When the temperature increases to 340 K, ${VO}_{2}$ transforms into its metallic state with $\sigma_{{\text{VO}}_{2}}=2\times 10^{5}$ S/m [6]. In this state, the ${VO}_{2}$ grating embedded in the substrate functions as a polarizer, providing high transmission for the y‐polarized wave and high reflection for the x‐polarized wave across a broad frequency band [34]. Detailed transmission and reflection spectra are provided in Supporting Information S1: Figure S1b in Note S1. Because of forward‐incident LP waves being first efficiently converted into their orthogonal polarization by the Ge dielectric pillars and then interacting with the ${VO}_{2}$ grating, their transmission behavior differs remarkably from the case of backward incidence, where the wave impinges directly on the ${VO}_{2}$ grating. The contrast is shown in Figure 6. Figure 6a,b presents the co‐ and cross‐polarized transmission spectra under forward and backward incidence of LP waves, respectively. Firstly, for the x‐polarized wave under forward incidence, the co‐polarized component vanishes across the entire frequency band of 2 THz−7 THz, whereas the cross‐polarized component exceeds 0.8 in the range of 3.7 THz–5.86 THz, with a maximum of 0.963 at 5 THz, demonstrating near‐perfect polarization conversion, as shown by the black and blue curves in Figure 6a. Under backward incidence, both of the transmittances of co‐ and cross‐polarized waves remain close to zero throughout the 2‐THz–7‐THz band, appearing as two flat lines, as shown by the black and blue curves in Figure 6b. Clearly, the transmission of x‐polarized waves exhibits significant asymmetry between the two propagation directions. The AT parameter for the x‐polarized wave $\left(\Delta_{\text{lin}}^{x}\right)$, calculated from Equation (7a), is plotted and indicated as the blue curve in Figure 6c. The results show that $\Delta_{\text{lin}}^{x}>0.8$ across the broadband of 3.7 THz–5.86 THz (gray‐shaded region), with a maximum of 0.958 at 5 THz. Clearly, at higher temperatures, the proposed TMPMM can function as a high‐performance broadband optical isolator, efficiently transmitting the forward‐incident x‐polarized wave while almost completely blocking the backward‐incident counterpart.

**FIGURE 6:**
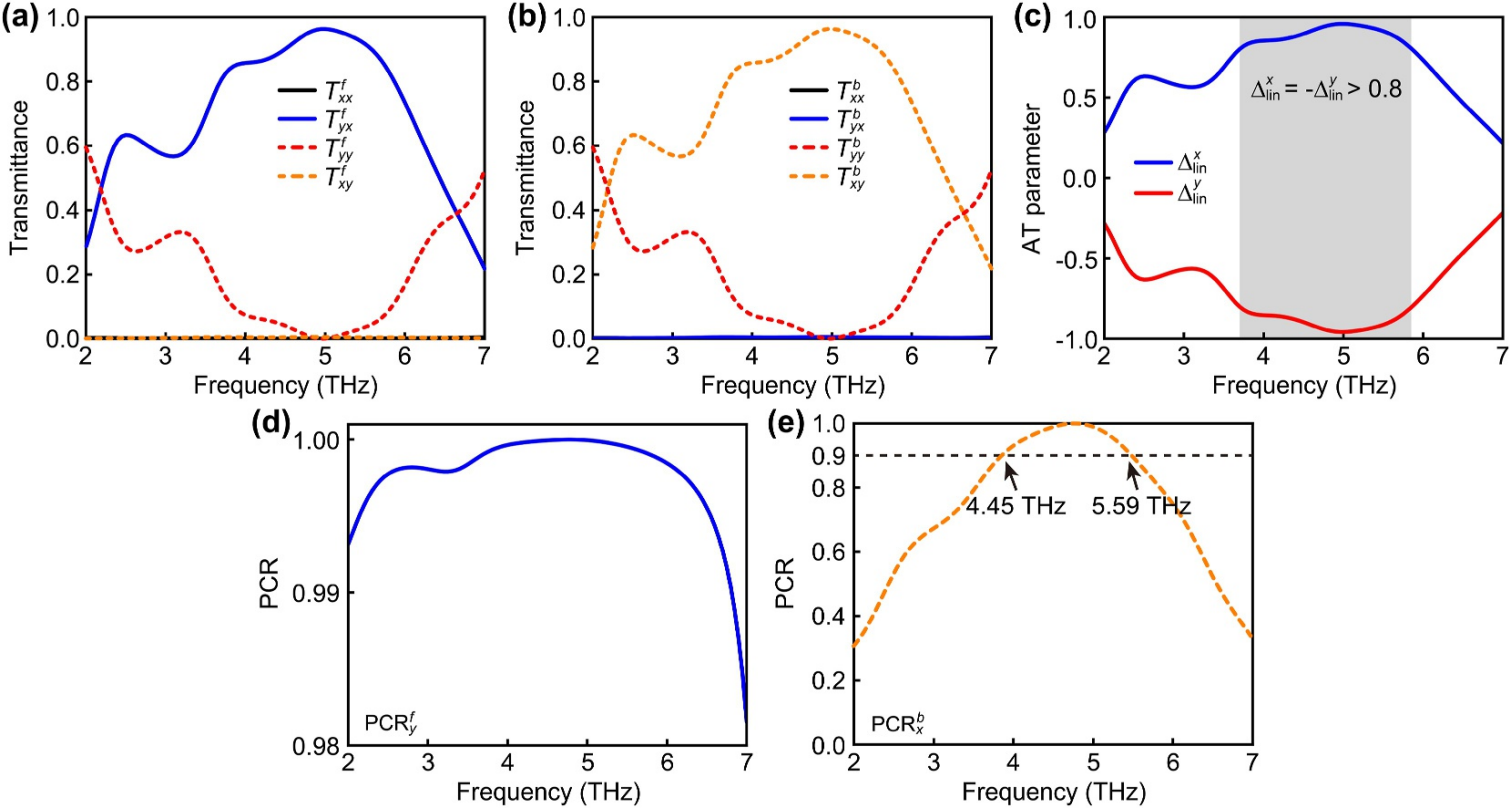
Co‐ and cross‐polarized transmission spectra for (a) forward‐ and (b) backward‐incident x‐ and y‐polarized waves at higher temperatures, respectively. (c) AT parameters. PCR under (d) forward and (e) backward incidence, respectively.

Next, consider the case of y‐polarized incidence. Under forward incidence, the cross‐polarized transmittance remains nearly zero across the entire 2 THz–7 THz frequency band, whereas the co‐polarized transmittance approaches zero near 5 THz and increases as the frequency deviates from this frequency, as shown by the orange and red dashed curves in Figure 6a. This phenomenon arises because the Ge dielectric pillars efficiently convert the incident y‐polarized wave into an x‐polarized wave over a broadband frequency range centered at 4.8 THz, resulting in the x‐polarized wave being reflected back by the ${VO}_{2}$ grating. As the frequency shifts outside the conversion band, the conversion efficiency gradually decreases, allowing the unconverted y‐polarized component to transmit through the TMPMM. Under backward incidence, the polarization modulation effect of the TMPMM primarily depends on the germanium dielectric pillars because the ${VO}_{2}$ grating is highly transparent to the y‐polarized wave. Consequently, the co‐polarized transmittance approaches zero around 5 THz, whereas the cross‐polarized transmittance reaches the maximum, as indicated by the red and orange dashed curves in Figure 6b. The AT parameter for the y‐polarized wave $\left(\Delta_{\text{lin}}^{y}\right)$, calculated from Equation (7b), is plotted as the red curve in Figure 6c. It can be seen that, over the broadband of 3.7 THz–5.86 THz (gray‐shaded region), $\Delta_{\text{lin}}^{y}<-0.8$, with a minimum of −0.958 at 5 THz, which is the opposite of $\Delta_{\text{lin}}^{x}$. Hence, for a y‐polarized wave, the proposed TMPMM achieves broadband high AT. Notably, around 5 THz, it only efficiently transmits a backward‐incident y‐polarized wave while almost completely blocking the forward‐incident counterpart, demonstrating its potential application as a narrowband optical isolator.

Figure 6d,e presents the PCR spectra for forward‐incident x‐polarized and backward‐incident y‐polarized waves, respectively. As shown in Figure 6d, for the forward‐incident x‐polarized wave, ${PCR}_{y}^{f}$ exceeds 0.98 across the entire frequency band. Furthermore, within the frequency range of 3.7 THz–5.86 THz, where $T_{yx}^{f}>0.8$ and ${PCR}_{y}^{f}>0.998$, indicating that the TMPMM achieves nearly perfect polarization conversion for the forward‐incident x‐polarized wave. For the backward‐incident y‐polarized wave, ${PCR}_{x}^{b}>0.9$ across the 4.45‐THz–5.59‐THz range, demonstrating that the TMPMM can efficiently convert the backward‐incident y‐polarized wave into an x‐polarized wave within the spectral region.

Additionally, the proposed TMPMM could also function as an optical switch for polarization conversion under the forward‐incident y‐polarized wave. As shown in Figure 7a, the blue and red curves represent the cross‐polarized transmission spectra at 300 and 340 K, respectively. At 300 K, the TMPMM efficiently converts the y‐polarized wave into an x‐polarized wave over the frequency range of 4.78 THz–5.17 THz, with a transmittance of $T_{xy}^{f}>0.958$, indicating that the switch is in the “ON” state. When the temperature rises to 340 K, $T_{xy}^{f}$ is suppressed below 0.01, turning the switch to the “OFF” state. To quantitatively evaluate the switching performance, the corresponding modulation depth (m) and extinction ratio (ER) are shown in Figure 7b, which are defined as follows:

(12) $$m=T_{\text{ON}}-T_{\text{OFF}}$$

(13) $$ER=10log\left(T_{\text{ON}}/T_{\text{OFF}}\right)$$

Here, $T_{\text{ON}}$ and $T_{\text{OFF}}$ denote the transmittance of the TMPMM at 300 and 340 K, respectively. Over the frequency band of 4.78–5.17 THz, m>0.948 and ER>20 dB. Specifically, at 4.95 THz, m reaches 0.98, and ER attains 36.95 dB, fully demonstrating an excellent switching capability of the proposed TMPMM.

**FIGURE 7:**
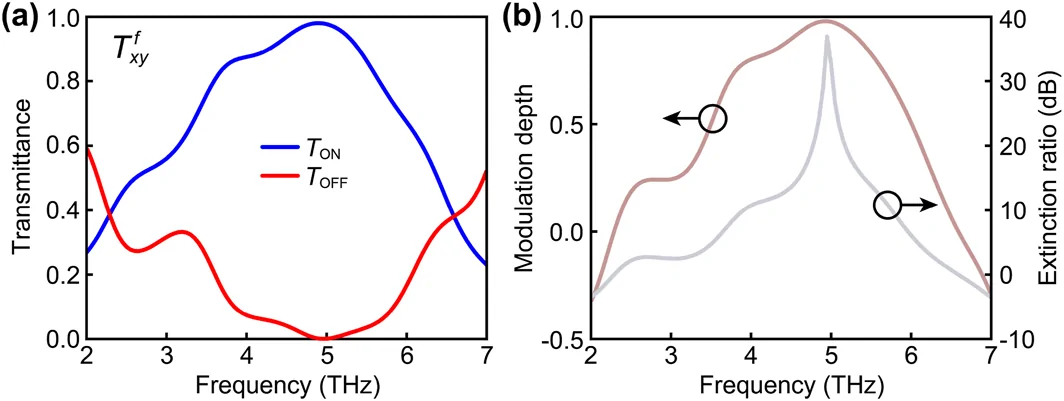
Temperature‐dependent polarization conversion transmittance and switching performance. (a) Polarization conversion transmittance of the TMPMM in the ON state (blue curve) and OFF state (red curve). (b) Corresponding modulation depth and extinction ratio spectra.

To gain deeper insights into the AT mechanism of the TMPMM at 340 K, a detailed analysis is performed using the Jones matrix. The amplitude and phase difference distributions of the transmission coefficients with u‐ and v‐polarized waves incident at this temperature are shown in Figure 8a,b, respectively. Over the broad frequency range of 2 THz–7 THz, the following approximate relations are found.

(14a) $$a_{uu}+a_{uv}\approx a_{vv}+a_{vu}$$

(14b) $$\Delta \Phi_{1}\approx \Delta \Phi_{2}$$

(14c) $$\Delta \Phi_{3}\approx 0$$

**FIGURE 8:**
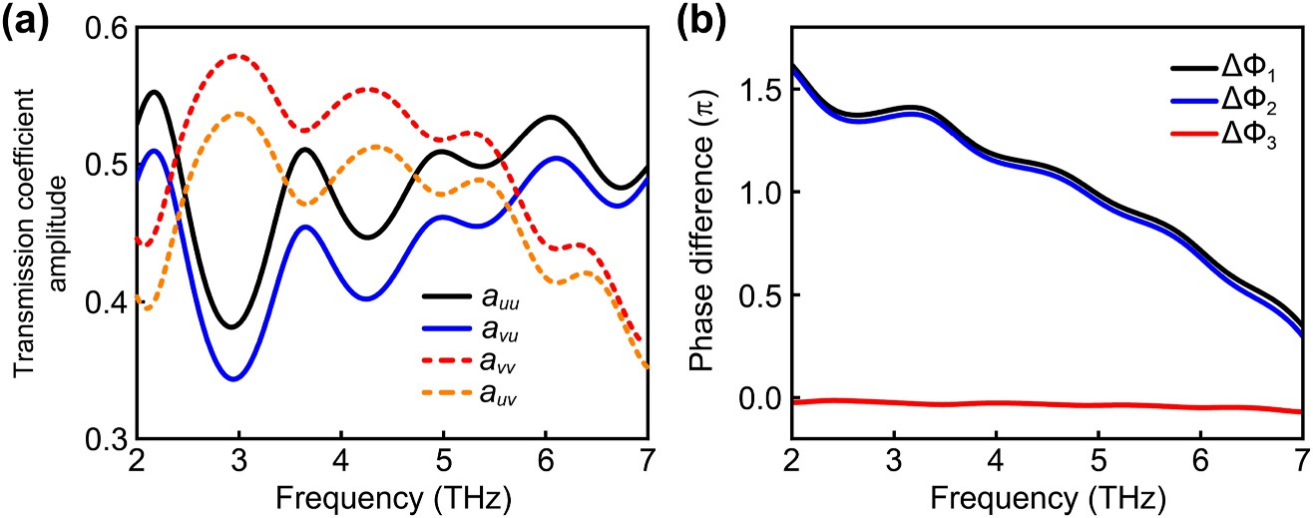
(a) Co‐polarized (black solid and red dashed) and cross‐polarized (blue solid and orange dashed) transmission coefficients with u‐ and v‐polarized incidence at higher temperatures. (b) Corresponding phase differences between $t_{uu}$ and $t_{vv}$ (black curve), $t_{vu}$ and $t_{vv}$ (blue curve), and $t_{uv}$ and $t_{vv}$ (red curve).

Consequently, for forward‐ and backward‐incident x‐polarized waves, the transmitted electric fields can be approximately expressed as follows:

(15) $$\left(\begin{array}{c} E_{x}^{t\left(f\right)}\\ E_{y}^{t\left(f\right)} \end{array}\right)\frac{1}{2}e^{i\Phi_{vv}}\left[\begin{array}{c} \left(a_{uu}-a_{vu}\right)\left(e^{i\Delta \Phi_{1}}+1\right)\\ e^{i\Delta \Phi_{1}}\left(a_{uu}+a_{vu}\right)-\left(a_{uv}+a_{vv}\right) \end{array}\right]$$

(16) $$\left(\begin{array}{c} E_{x}^{t\left(b\right)}\\ E_{y}^{t\left(b\right)} \end{array}\right)=\frac{1}{2}e^{i\Phi_{vv}}\left[\begin{array}{c} \left(a_{uu}-a_{vu}\right)\left(e^{i\Delta \Phi_{1}}+1\right)\\ \left(a_{uu}-a_{vu}\right)\left(e^{i\Delta \Phi_{1}}-1\right) \end{array}\right]$$

According to Equations (15) and (16), within the broadband frequency band of 2–7 THz, because the term $a_{uu}-a_{vu}$ remains consistently small, when $e^{i\Delta \Phi_{1}}$ varies, both $|t_{xx}^{f}|$, $|t_{xx}^{b}|$, and $|t_{yx}^{b}|$ are remaining close to zero, whereas $|t_{yx}^{f}|$ reaches a near‐unity peak when $\Delta \Phi_{1}=\pi$.

As shown in Figure 8b, $\Delta \Phi_{1}$ decreases from 1.615π at 2 THz to 0.348π at 7 THz, approaching π at 4.95 THz. Thus, the proposed TMPMM exhibits an AT close to 1 for the x‐polarized wave around 4.95 THz. Moreover, over the entire 2 THz–7 THz frequency band, $|t_{yx}^{f}|\gg |t_{xx}^{f}|$, indicating that the forward‐incident x‐polarized wave is efficiently converted into a y‐polarized wave, whereas the backward‐incident x‐polarized wave is almost completely blocked. This confirms that the structure can serve as a high‐performance broadband optical isolator. The frequency of 4.95 THz obtained here is consistent with 5 THz, as shown in Figure 6; the slight deviation of 0.05 THz arises from approximations in the amplitudes and phases. A detailed analysis of the mechanisms of the AT for the y‐polarized wave is provided in Supporting Information S1: Note S5. The corresponding electric field distributions are shown in Supporting Information S1: Note S6.

### 3.3 Spin‐Dependent Polarization Selection

As discussed in Section 3.1, the Ge dielectric pillars function as QWPs in both the low‐frequency and high‐frequency regions. When CP waves of opposite polarizations are incident from the forward direction, they are transformed into orthogonal LP waves in the low‐frequency and high‐frequency regions, respectively. Additionally, at 340 K, the ${VO}_{2}$ grating functions as a linear polarization selector. Obviously, in both the low‐frequency and high‐frequency regions, CP waves with different polarizations are efficiently transmitted or reflected after interacting with the TMPMM, achieving spin‐dependent polarization selection. To validate this analysis, the simulated transmission and reflection spectra for RCP and LCP waves under forward incidence are shown in Figure 9a,b, respectively. In the low‐frequency region near 2.25 THz, for an incident RCP wave, the peak reflectance for a co‐polarized wave reaches 0.836, whereas the cross‐polarized reflectance, co‐polarized transmittance, and cross‐polarized transmittance all remain below 0.014. This indicates that the incident RCP wave is efficiently reflected while preserving its helicity. For the incident LCP wave, the co‐ and cross‐polarized reflectances are only 0.043 and 0.01, respectively, whereas the co‐ and cross‐polarized transmittances are 0.459 and 0.471, respectively. This indicates that the incident LCP wave is efficiently transmitted and converted into an LP wave, as illustrated by the orange curve in Figure 9c. In the high‐frequency region near 6.37 THz, for an incident RCP wave, the reflectances are low (0.083 for co‐polarized and 0.032 for cross‐polarized), whereas both transmittances of co‐ and cross‐polarized waves are approximately equal to 0.427. This indicates that the incident RCP wave is efficiently transmitted and converted into an LP wave, as shown by the blue curve in Figure 9c. For an incident LCP wave, the co‐polarized reflectance reaches 0.798, whereas the corresponding cross‐polarized reflectance, co‐polarized transmittance, and cross‐polarized transmittance are all below 0.033, indicating that the incident LCP wave is efficiently reflected while preserving its helicity. The proposed TMPMM selectively reflects the RCP wave in the low‐frequency region and the LCP wave in the high‐frequency region, exhibiting remarkable spin‐dependent polarization selection. To provide an intuitive description of the differences between RCP and LCP waves in reflection and transmission, Figure 9d,e further displays the CD spectra of the TMPMM in reflection and transmission at different frequencies, defined as follows:

(17) $$CD_{R}=R_{+-}^{f}+R_{--}^{f}-R_{-+}^{f}-R_{++}^{f}=R_{\mathrm{LCP}}-R_{\mathrm{RCP}}$$

(18) $$CD_{T}=T_{+-}^{f}+T_{--}^{f}-T_{-+}^{f}-T_{++}^{f}=T_{\mathrm{LCP}}-T_{\mathrm{RCP}}$$

Here, $R_{\mathrm{RCP}}$ and $R_{\mathrm{LCP}}$ denote the total reflectance under forward‐incident RCP and LCP waves, respectively, whereas $T_{\mathrm{RCP}}$ and $T_{\mathrm{LCP}}$ denote the total transmittance under forward‐incident RCP and LCP waves, respectively. For ${CD}_{R}$, it can be observed that, within the dual bandwidths of 0.83 THz–3.72 THz and 6.06 THz–6.67 THz, $\left|{CD}_{R}\right|>0.6$. Additionally, strong chiral responses are observed at specific frequencies, with values of −0.796 at 1.1 THz, −0.789 at 2.25 THz, −0.748 at 3.38 THz, and 0.715 at 6.37 THz. For ${CD}_{T}$, it can be observed that within the dual bandwidths of 0.86 THz–3.7 THz and 6.03 THz–6.83 THz. Additionally, strong chiral responses are observed at specific frequencies, with values of 0.853 at 1.1 THz, 0.917 at 2.27 THz, 0.855 at 3.4 THz, and −0.83 at 6.36 THz.

**FIGURE 9:**
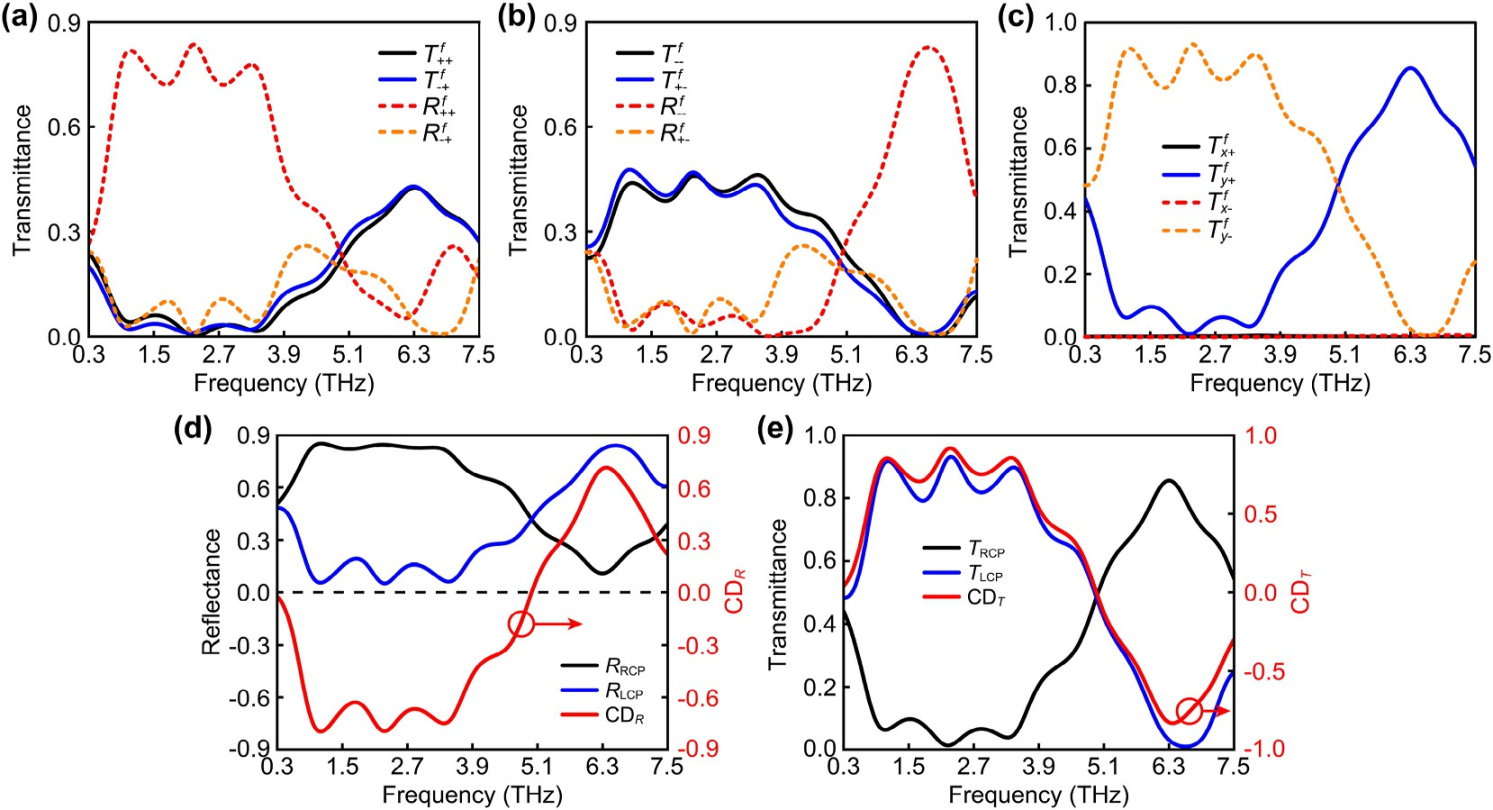
Spin‐dependent polarization selection results. Co‐ and cross‐polarized transmittance and reflectance spectra after forward‐incident (a) RCP and (b) LCP wave illumination of the TMPMM. (c) LP components in transmission. (d) Total reflectance under forward‐incident RCP (black curve) and LCP (blue curve) waves and their reflectance difference (red curve). (e) Total transmittance under forward‐incident RCP (black curve) and LCP (blue curve) waves and their transmittance difference (red curve).

Notably, because LP waves of any polarization state can be expressed as a linear superposition of RCP and LCP components, the TMPMM efficiently reflects RCP waves (while preserving their chirality) and exhibits near‐zero reflectivity for LCP waves at approximately 2.25 THz. Therefore, at this frequency, the structure can effectively convert LP waves of arbitrary polarization into RCP waves. In transmission, the TMPMM efficiently transmits LCP waves and converts them into y‐polarized waves, while exhibiting near‐zero transmittance for RCP waves at approximately 2.25 THz. Meanwhile, benefiting from the polarization‐selective characteristics of the grating, RCP waves incident at other frequencies are also converted into y‐polarized waves in transmission. Therefore, over a broad frequency range around this frequency, the structure can convert LP waves of arbitrary polarization into near‐perfect y‐polarized waves. A detailed discussion is provided in Supporting Information S1: Note S7. This conversion mechanism, based on spin‐dependent polarization selection, differs distinctly from previous reports that require specific incident polarization directions [50, 51, 52]. The findings here offer a novel approach for constructing robust CP/LP wave generators with low sensitivity to polarization angle. Furthermore, through structural optimization, the chiral response can be further enhanced, as discussed in detail in Supporting Information S1: Note S8.

### 3.4 Various Dynamic Controls of AT, CD and Ellipticity With Conductivity Changes

Next, systematic investigation of the three tunable properties enabled by the conductivity variation of ${VO}_{2}$ (corresponding to variations in temperature) for device functionality is performed, including dynamic control of AT, CD, and ellipticity. All these tunable properties confirm that the proposed TMPMM serves as an effective platform for dynamic polarization manipulation.

#### 3.4.1 Dynamic Control of AT

Figure 10a shows the variation trend of the AT parameters with different conductivities of ${VO}_{2}$. It can be observed that, within the frequency range of 3.7 THz–5.86 THz, as $\sigma_{{\text{VO}}_{2}}$ increases from 20 S/m to $2\times 10^{5}$ S/m, the magnitude of the AT parameter effectively rises from near zero to above 0.8. This demonstrates that the AT efficiency can be flexibly controlled by tuning the conductivity. Specifically, at 5 THz, the AT parameter increases from 0.001 to 0.958, yielding a modulation depth as high as 0.957, which highlights the significant advantage of the proposed structure for achieving high‐contrast AT switching. It is worth emphasizing that, although previous studies have realized dynamic tuning of the AT parameter by integrating bilayer metallic gratings with tunable materials, all of these approaches typically come at the cost of sacrificing forward transmittance [30, 53]. In particular, when the AT parameter in those structures is tuned to zero, both forward‐ and backward‐incident waves are reflected, and transmission is strongly suppressed, limiting the utilization efficiency of the system's energy. For comparison, Figure 10b presents the cross‐polarized transmittance of the TMPMM under different $\sigma_{{\text{VO}}_{2}}$. It is observed that, as $\sigma_{{\text{VO}}_{2}}$ increases, the overall transmittance first decreases and then increases, reaching its minimum at $\sigma_{{\text{VO}}_{2}}=5\times 10^{3}$ S/m. Nevertheless, around 5 THz, the cross‐polarized transmittance remains above 0.8 for all the examined conductivities, as indicated by the black dashed line in Figure 10b. Accordingly, the proposed TMPMM not only enables dynamic control of AT but also maintains high‐efficiency transmission, a feature that is crucial for practical applications such as communication and imaging [28, 29].

**FIGURE 10:**
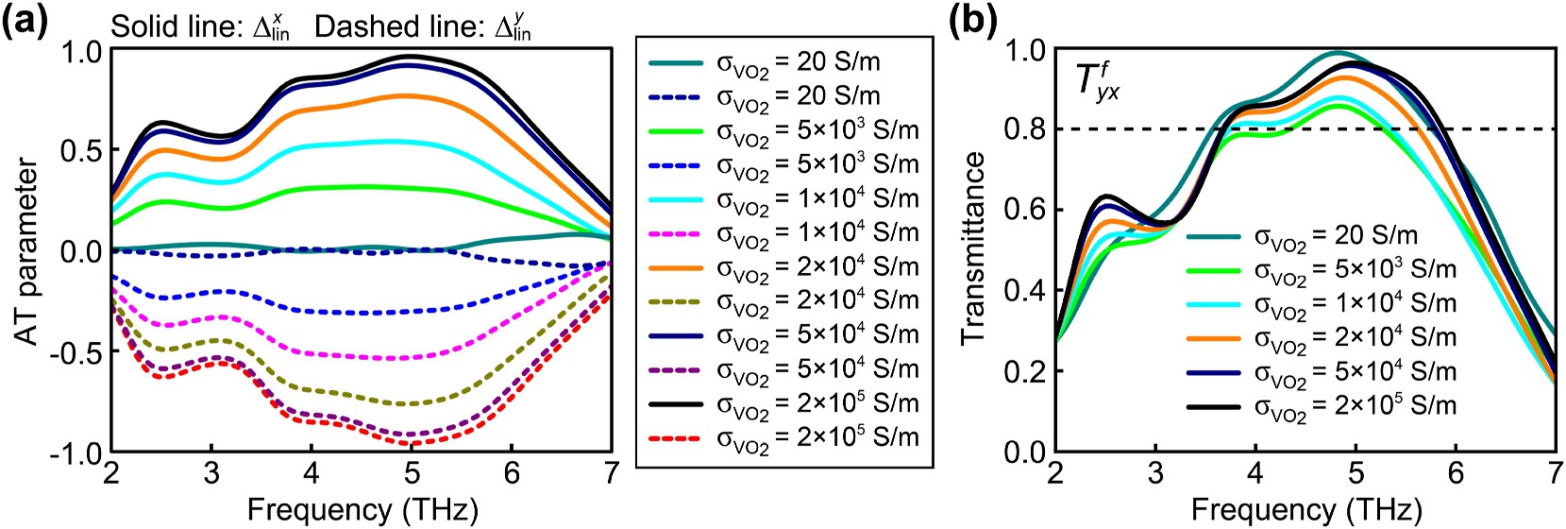
Dynamic control of AT. (a) AT parameters with different $\sigma_{{\text{VO}}_{2}}$. Solid and dashed curves correspond to x‐ and y‐polarized incidence, respectively. (b) Cross‐polarized transmittance for forward‐incident x‐polarized waves with different ${VO}_{2}$ conductivities.

#### 3.4.2 Dynamic Control of CD

Dynamic control of the chiral response holds significant potential for applications such as polarization manipulation, sensing, and imaging [54]. However, modifying chiral effects often faces challenges because the chiral response mainly relies on the structural parameters of the metasurface, which are difficult to adjust after fabrication [55, 56]. To address this issue, this work leverages the tunable conductivity of $VO_{2}$ in the proposed TMPMM and presents an effective method for achieving dynamic control of CD. The dynamically tunable performance of ${CD}_{T}$ is illustrated in Figure 11. Figure 11a shows the ${CD}_{T}$ spectra at different $\sigma_{{VO}_{2}}$, whereas Figure 11b presents the corresponding transmittance difference of the $VO_{2}$ grating for x‐ and y‐polarized waves. When $\sigma_{{VO}_{2}}=20$ S/m, the $VO_{2}$ grating exhibits nearly identical transmission modulation for x‐ and y‐polarized waves across the frequency range of 0.3 THz–6.26 THz, with a transmittance difference below 0.05 (black curve in Figure 11b). Consequently, the transmittance with RCP and LCP incidence is mostly the same, resulting in an overall nonchiral response. At frequencies above 6.26 THz, the transmittance of the $VO_{2}$ grating for the y‐polarized wave gradually exceeds that for the x‐polarized wave. At 6.8 THz, because the incident RCP wave is mainly converted into a y‐polarized wave by the Ge dielectric pillars, whereas the LCP wave is primarily converted into an x‐polarized wave, the transmittance difference of −0.144 between the two polarizations leads to a corresponding difference in the transmitted RCP and LCP waves, generating a weak ${CD}_{T}$ response of −0.129, as shown by the black curve in Figure 11a. As $\sigma_{{VO}_{2}}$ gradually increases, the transmittance difference between x‐ and y‐polarized waves in the $VO_{2}$ grating exhibits an increasing trend, effectively enhancing the ${CD}_{T}$ response. When $\sigma_{{VO}_{2}}$ increases from 20 S/m to $2\times 10^{5}$ S/m, at 2.27 THz and 6.36 THz, ${CD}_{T}$ is tuned from 0.021 and −0.07 to 0.917 and −0.83, respectively, with corresponding maximum modulation depths $\Delta {CD}_{T}$ reaching 0.896 and −0.76. Ultimately, by tuning the ${VO}_{2}$ conductivity, ${CD}_{T}$ can be transformed from a nearly zero nonchiral response to a strong chiral response exceeding 0.7 across the dual broadband ranges of 0.86 THz–3.7 THz and 6.03 THz–6.83 THz.

**FIGURE 11:**
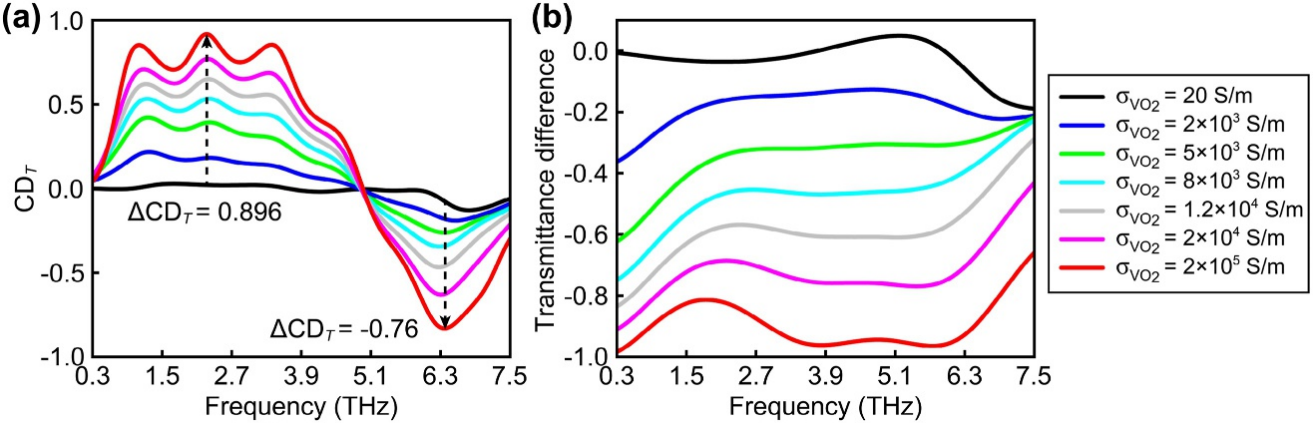
Dynamic control of $CD_{T}$. (a) $CD_{T}$ spectra under different $\sigma_{{\text{VO}}_{2}}$. (b) Transmittance difference between x‐ and y‐polarized waves for the $\sigma_{{\text{VO}}_{2}}$ grating at corresponding conductivities.

#### 3.4.3 Dynamic Control of Ellipticity

In addition to enabling dynamic control of AT and CD, the proposed TMPMM structure also offers continuous control over the polarization state of the transmitted waves, allowing on‐demand output of CP, elliptically polarized (EP), or LP waves. Figure 12a,b shows the ellipticity η of the transmitted wave as a function of $\sigma_{{VO}_{2}}$ for x‐ and y‐polarized incidence, respectively. The results demonstrate that, within the frequency range of 2.31 THz–2.58 THz, as $\sigma_{{VO}_{2}}$ increases from 20 S/m to $2\times 10^{5}$ S/m, the η of the forward‐incident x‐polarized wave can be effectively tuned from above 0.9 to below 0.1, whereas that for forward‐incident y‐polarized waves shifts from below −0.9 to above −0.1. This demonstrates a continuous transition from RCP/LCP waves toward LP waves, respectively. Figure 12c,d presents the polarization azimuth angle φ (where $\varphi =\left[\tan^{-1}\left(S_{2}/S_{1}\right)\right]/2$ [27]) of the transmitted wave as a function of $\sigma_{{VO}_{2}}$ for forward‐incident x‐ and y‐polarized waves, respectively. It can be observed that the drastic changes in φ primarily occur within the range of small $\sigma_{{VO}_{2}}$, whereas φ approaches 90° for both incidence cases as $\sigma_{{VO}_{2}}$ further increases. The drastic changes in φ in the low‐conductivity range occur because the transmitted waves in this range approximate ideal CP waves (particularly under y‐polarized incidence), and because φ of an ideal CP wave cannot be defined, even minor variations in electric field amplitude and phase can lead to significant changes in φ. For this reason, in Figure 12d, φ variations corresponding to η≈1 are partially omitted to prevent the excessively wide range of variation from obscuring the trend of φ in other regions. When $\sigma_{{VO}_{2}}$ is large, the polarization‐selective properties of the grating allow only the transmission of the y‐polarized component, causing φ to gradually approach 90°. To quantitatively describe the evolution of the polarization states, Figure 12e,f presents the amplitudes of η and φ at 2.45 THz under different $\sigma_{{VO}_{2}}$, respectively. When $\sigma_{{VO}_{2}}=20$ S/m, the ellipticities after x‐ and y‐polarized incident waves reach as high as −0.925 and 0.996, respectively, indicating that the incident waves are efficiently converted into LCP and RCP waves. When $\sigma_{{VO}_{2}}=2\times 10^{5}$ S/m, the corresponding ellipticities decrease to as low as −0.052 and 0.075, respectively, demonstrating that the transmitted waves have evolved into LP waves. Therefore, the continuous transition of η from near 1/−1 to around 0 in Figure 12e fully demonstrates the capability of the TMPMM to achieve complete polarization control. Regarding the polarization azimuth angle φ, opposite trends are observed for x‐ and y‐polarized incident waves, as shown by the blue and red curves in Figure 12f. As $\sigma_{{VO}_{2}}$ increases, the φ for x‐polarized incidence first increases and then decreases to approximately 90°, whereas φ for y‐polarized incidence first decreases and then increases to around 90°. Figure 12g further plots the evolution of the polarization state of the transmitted wave on the Poincaré sphere. It can be seen that as $\sigma_{{VO}_{2}}$ increases, the polarization trajectories for x‐ and y‐polarized incident waves converge from the two poles of the sphere toward the equator, respectively, intuitively revealing the transition of the transmitted wave from CP to LP, which is in excellent agreement with the previous analysis. Figure 12h displays representative polarization states at 2.45 THz for different $\sigma_{{VO}_{2}}$. When $\sigma_{{VO}_{2}}=20$ S/m, $\sigma_{{VO}_{2}}=2\times 10^{4}$ S/m, and $\sigma_{{VO}_{2}}=2\times 10^{5}$ S/m, the incident x‐polarized wave can be converted into LCP, LEP, and y‐polarized waves, respectively, whereas the incident y‐polarized wave can be converted into RCP, REP, and y‐polarized waves, respectively. Thus, by dynamically tuning the conductivity of ${VO}_{2}$, the proposed TMPMM provides an effective route toward active and flexible polarization‐state manipulation.

**FIGURE 12:**
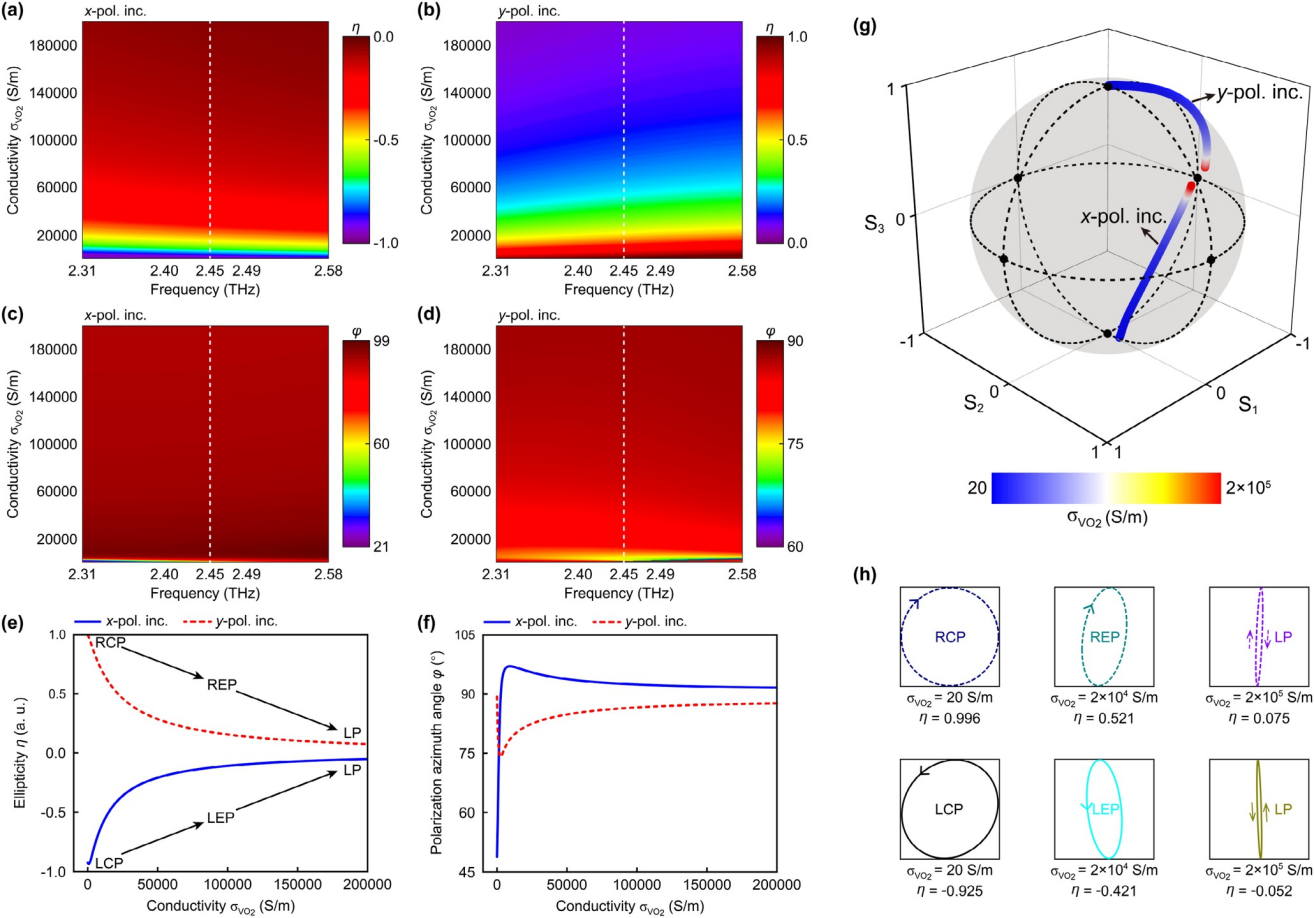
Polarization‐state control characteristics of the TMPMM for incident LP waves under different $\sigma_{{VO}_{2}}$. Ellipticity distributions for (a) x‐ and (b) y‐polarized incidence. Polarization azimuth angle variations for (c) x‐ and (d) y‐polarized incidence. At 2.45 THz, (e) η and (f) φ as functions of $\sigma_{{VO}_{2}}$. (g) The evolution of the corresponding Stokes parameters on the Poincaré sphere. (h) Representative polarization states.

Furthermore, over a broad frequency range of 4.71 THz–4.89 THz, the structure also enables continuous tuning of ellipticity under RCP and LCP incidence. By further combining metasurface rotation, the TMPMM can achieve full‐Stokes polarization control without relying on frequency multiplexing; see Supporting Information S1: Note S9 for a detailed discussion.

### 3.5 Performance Comparison

To objectively evaluate the polarization‐manipulation capability of the proposed TMPMM in the terahertz regime, a comprehensive performance comparison with existing relevant studies is conducted, and the results are summarized in Table 1. For a unified presentation, the polarization conversion efficiency of the metasurface functioning as an HWP is expressed in terms of $T_{\text{cross}}$ and $R_{\text{cross}}$, whereas that of a QWP is expressed as $T_{\mathrm{RCP}}$ and $T_{\mathrm{LCP}}$ in the table. Compared with single‐function polarization converters [17, 57], the TMPMM demonstrates superior performance in both operational bandwidth and conversion efficiency. Furthermore, compared to polarization‐control devices that also integrate tunable materials [5, 6, 30, 32, 52], our design not only provides a broader operating band and higher conversion efficiency but also enables more comprehensive polarization‐control functionalities, including HWP, QWP, AT, polarization conversion switch, and CD. Notably, by simply tuning the conductivity of ${VO}_{2}$, the TMPMM can simultaneously realize dynamic control of AT, CD, and ellipticity. It should be noted that the results presented in this work are obtained through numerical simulations, whereas some of the comparator devices in Table 1 have been experimentally demonstrated. To ensure consistency in the performance comparison, all metrics listed in Table 1 are based on results from numerical simulations.

**TABLE 1:** Performance comparison of THz metasurface devices.

Year (ref.)	HWP	QWP	AT	CD	Controllable
2019 [30]	0.2–1.6 THz	—	0.2–2 THz	—	Tuning AT by changing the
	PCR>0.99		AT≤0.7		Fermi energy of graphene
	$T_{\text{cross}}<0.8$				
2021 [57]	0.219–0.334 THz	—	—	—	—
	$T_{\text{cross}}>0.77$				
2022 [6]	0.61–1.63 THz	—	0.64 THz	—	Switch HWP and AT by
	PCR>0.9		AT=0.7		changing the phase of ${VO}_{2}$
	$R_{\text{cross}}<0.8$		1.28 THz		
			AT=0.7		
2023 [27]	0.61–0.94 THz	—	0.3–1.2 THz	0.25–0.52 THz	Control multifunction by
	PCR>0.8		AT<0.6	CD>0	changing the incident waves
	$T_{\text{cross}}<0.6$			$CD_{\text{max}}\approx 0.64$	polarization state
2023 [5]	0.44–0.9 THz	—	0.42 THz	—	Switch HWP and AT by
	PCR>0.87		AT=0.61		changing the phase of ${VO}_{2}$
	$R_{\text{cross}}<0.8$		0.81 THz		
			AT=0.65		
2024 [32]	—	—	AT for dual band	—	Switchable single‐ and
			AT≈0.83 at 3.57 THz		Dual‐band AT by
			AT≈0.86 at 5.91 THz		changing the phase of ${VO}_{2}$
			AT for single band		
			AT≈0.58 at 3.77 THz		
2025 [52]	0.88–1.78 THz	0.81–1.26 THz	—	—	Switch HWP and QWP by
	PCR>0.9	$\left|\eta \right|>0.91$			changing the phase of ${VO}_{2}$
	$R_{\text{cross}}<0.85$	$T_{RCP\left(max\right)}=0.91$			
		$T_{LCP\left(max\right)}=0.91$			
2025 [17]	0.53–1.53 THz	—	—	—	—
	PCR>0.9				
**This work**	**300 K: 3.88–5.5 THz**	**2.31–2.61 THz**	**3.7–5.86 THz**	**0.83–3.72 THz**	**Dynamically control AT,**
	PCR>0.9	$\left|\eta \right|>0.9$	AT>0.8	CD<−0.6	**CD, and ellipticity by**
	$T_{\text{cross}}>0.86$	$T_{RCP\left(max\right)}=0.917$	$AT_{\text{max}}=0.958$	$CD_{\text{max}}=-0.796$	**tuning** ${\text{VO}}_{2}$ **conductivity**
	***T***_**cross(max)**_ ≥ **0.98**	$T_{LCP\left(\text{max}\right)}=0.937$		**6.06–6.76 THz**	
	**340 K: 3.68–5.87 THz**	**6.42–6.68 THz**		CD>0.6	
	PCR>0.998	$\left|\eta \right|>0.9$		$CD_{\text{max}}=0.715$	
	$T_{\text{cross}}>0.8$	$T_{RCP\left(max\right)}=0.954$			
	***T***_**cross(max)**_ = **0.963**	$T_{LCP\left(max\right)}=0.87$			

*Note:* The bold values highlight the superior performance of our proposed TMPMM structure compared with the other referenced work.

By uniformly scaling the geometric dimensions of the structure, the operating frequency of the proposed TMPMM can be effectively extended to several GHz. A detailed discussion is provided in Supporting Information S1: Note S10.

### 3.6 Potential Fabrication Methods of the TMPMM

The proposed metasurface structure can be fabricated using the process flow illustrated in Figure 13. The main steps are outlined as follows. The fabrication starts with a $SiO_{2}$ handle layer approximately 300 μm thick. First, a $VO_{2}$ thin film is deposited onto the $SiO_{2}$ handle layer via magnetron sputtering, and the $VO_{2}$ grating structure is formed through electron‐beam lithography and a lift‐off process [58, 59]. Subsequently, a 15‐μm‐thick $SiO_{2}$ layer is deposited via plasma‐enhanced chemical vapor deposition (PECVD) [60], and a Ge film is deposited onto the $SiO_{2}$ layer using high‐density plasma chemical vapor deposition (HDPCVD) [61, 62]. A chromium (Cr) film is then deposited on the Ge surface as a hard mask layer using the electron‐beam (E‐beam) deposition technique [36]. The Cr film is subsequently coated with photoresist. The structural features of the central dielectric pillars are exposed by E‐beam lithography (to facilitate observation of the multidielectric pillar characteristics, cross‐sectional views in the v‐z plane are used hereafter, where v represents the short‐axis direction of the Ge dielectric pillar). The nanopattern is transferred onto the $SiO_{2}$ mask using a lift‐off process. The pattern is then transferred to the Cr hard mask layer using an inductively coupled plasma‐reactive ion etching (ICP‐RIE) system [36]. The sample with the patterned Cr hard mask layer is etched by ICP to achieve the desired height difference between the central Ge pillar and the side pillars. A Cr film is then reapplied as a hard mask layer, followed by coating the Cr film with photoresist. The structural features of all pillars are exposed by E‐beam lithography. The nanopattern is transferred onto the $SiO_{2}$ mask using a lift‐off process, and the pattern is subsequently transferred to the Cr hard mask layer using ICP‐RIE. The Cr hard mask layer is then etched. The sample is etched by ICP to fabricate all Ge pillars. The patterned Cr hard mask is removed using a Cr etchant, yielding the top Ge dielectric pillars. Finally, the $SiO_{2}$ in the handle layer is etched by ICP to reduce the thickness beneath the corresponding region of the top Ge pillars to 30 μm. Meanwhile, the $SiO_{2}$ handle layer in the nonstructural region is retained to serve as a supporting structure [63], obtaining the final sample.

**FIGURE 13:**
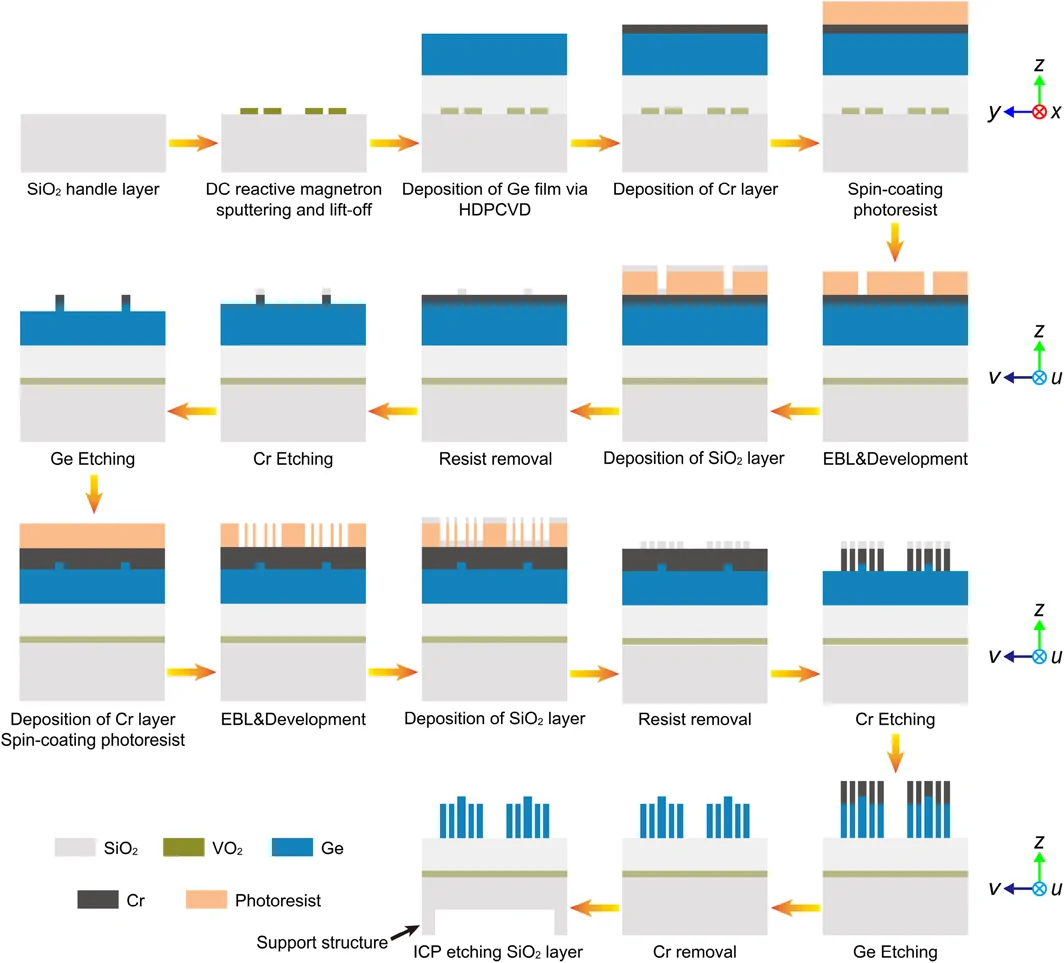
Schematic illustration of the potential fabrication process for the metasurface.

## 4 Conclusion

In summary, a TMPMM based on cascaded Ge dielectric pillars and a ${VO}_{2}$ grating is proposed. At room temperature (300 K), the structure achieves efficient cross‐polarization conversion within a broad bandwidth of 3.88 THz–5.5 THz for both forward and backward incidence of LP waves, with an overall orthogonal polarization transmittance above 0.86 and PCRs exceeding 0.9, serving as an efficient broadband HWP. Within the frequency ranges of 2.31 THz–2.61 THz and 6.42 THz–6.68 THz, a backward‐incident x‐polarized (y‐polarized) wave is efficiently converted into RCP (LCP) and LCP (RCP) waves, respectively, with the ellipticity consistently remaining above 0.9. The maximum conversion efficiencies reach 0.917 (0.937) and 0.87 (0.954), demonstrating functionality as a high‐performance QWP. At high temperatures (340 K), the ${VO}_{2}$ grating exhibits high transmission for x‐polarized waves and high reflection for y‐polarized waves, thereby generating a significant AT effect for forward‐ and backward‐incident LP waves. This effect enables broadband isolators and an HWP for x‐polarized waves, as well as narrowband isolators and polarization conversion optical switches for y‐polarized waves. When forward‐incident RCP and LCP waves are considered, the structure selectively transmits LCP and RCP waves near 2.25 THz and 6.37 THz, respectively, demonstrating remarkable spin‐dependent polarization selectivity. This provides a novel approach for constructing robust CP/LP wave generators. Through Jones matrix analysis and multipole decomposition, the physical mechanisms underlying these polarization controls are systematically elucidated. Furthermore, by leveraging the temperature‐controlled conductivity of ${VO}_{2}$, the proposed TMPMM enables dynamic control of AT, CD, and ellipticity, allowing for flexible switching between symmetric and asymmetric transmission, transitions from nonchiral to strongly chiral responses, and continuous modulation between CP and LP waves. Our work provides a valuable design strategy for realizing multifunctional, multiband, highly efficient, and dynamically tunable polarization manipulation devices, which is expected to advance the development of polarization devices and chiral functional devices in the field of integrated photonics.

## Author Contributions

**Xingxing Fan:** conceptualization, investigation, software, validation, visualization, writing – original draft, writing – review and editing. **Qiang Bian:** validation, resources, writing – review and editing, data curation. **Qingguo Du:** conceptualization, methodology, investigation, funding acquisition, visualization, project administration, resources, supervision, writing – review and editing, writing – original draft. **Song Sun:** writing – review and editing, validation, visualization, resources, funding acquisition. **Qin Fu:** methodology, software, formal analysis, writing – review and editing. **Zhengying Li:** investigation, project administration, resources, writing – review and editing, validation. **Fei Xu:** writing – review and editing, validation, formal analysis, data curation.

## Conflicts of Interest

The authors declare no conflicts of interest.

## Supporting information


Supporting Information S1

## Data Availability

The data that support the findings of this study are available from the corresponding author upon reasonable request.
