# Supplementary material for: Transmissive Terahertz Tunable and Multifunctional Polarization Manipulation With Ge/VO2 Hybrid Metasurface
Source: Nanophotonics. 2026 Jun 19;15(12):e70175. doi: 10.1002/nap2.70175 (PMC13642087; doi:10.1002/nap2.70175)
Supplement: Supplementary file 1 [file NAP2-15-e70175-s001.pdf]

Supporting Information

Transmissive terahertz tunable and multifunctional polarization manipulation with Ge/VO_2 hybrid metasurface

Xingxing Fan, Qiang Bian, Qingguo Du*, Song Sun*, Qin Fu, Zhengying Li,* and *Fei Xu*

1 Supplementary Note 1 Temperature-dependent modulation of the VO_2 grating

Figure S1(a) and (b) illustrate the co-polarized transmittance and reflectance of the VO_2 grating for x- and y-polarized waves at temperatures of 300 K and 340 K, respectively. This numerical calculation does not include the five Ge dielectric pillars on the top layer, while all other structural parameters remain consistent with the main text. As shown in Figure S1(a), at 300 K (VO_2 in insulating state), the transmittance and reflectance difference between x- and y-polarized waves is relatively small over the frequency range of 0.3 THz - 6.17 THz, manifested as $T_{xx} \approx T_{yy}$ and $R_{xx} \approx R_{yy}$. However, above 6.17 THz, the reflectance for the x-polarized wave gradually exceeds that for the y-polarized wave, and both the transmittance and reflectance differences increase with frequency. This trend is consistent with the transmission spectra observed in Figure 2 of the main text. When the temperature rises to 340 K (VO_2 in metallic state), the VO_2 grating exhibits a stable modulation response to y-polarized wave, maintaining high transmittance across the broad band of 3 THz - 6 THz, as indicated by the blue curve in Figure S1(b). In contrast, the x-polarized wave is mostly reflected rather than transmitted, exhibiting a significant reflection enhancement shown by the red dashed curve in Figure S1(b). These results demonstrate that the proposed VO_2 grating behaves approximately as a transparent medium at room temperature, while it functions as an efficient polarization-selective element at elevated temperatures. This property provides the foundation for achieving temperature-dependent dynamic control.

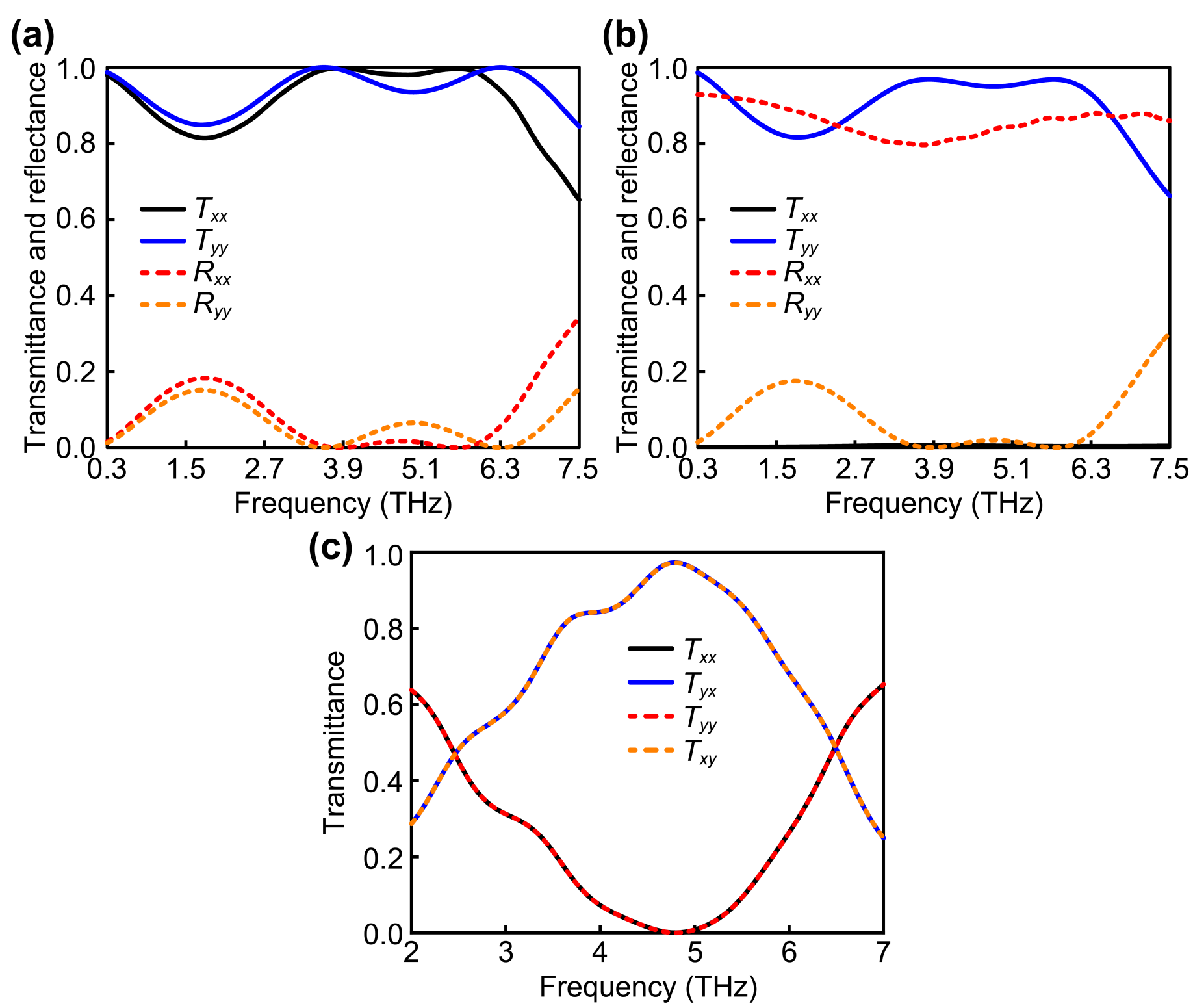

Figure S1. Co-polarized transmittance and reflectance of the VO_2 grating for x- and y-polarized waves at temperatures of (a) 300 K and (b) 340 K. (c) Co- and cross-polarized transmittance of TMPMM for x-polarized and y-polarized waves without VO_2 grating.

Furthermore, to visually demonstrate that the slight difference between the x-polarized and y-polarized waves in the TMPMM is caused by the VO_2 grating, the modulation effect without the VO_2 grating is simulated as shown in Figure S1(c). It is clearly observed that the co-polarized transmission and cross-polarized transmission of the x-polarized wave after passing through the TMPMM match those of the y-polarized wave incident on the system. This result confirms that the response difference between x- and y-polarized waves in the TMPMM originates from the VO_2 grating, validating the correctness of this analytical conclusion.

2 Supplementary Note 2 Control of circularly polarized waves by the TMPMM at room temperature and its advantages in multifunctional polarization manipulation

In addition to its capability to rotate the polarization direction of linearly polarized (LP) waves, another essential function of a half-wave plate (HWP) is to reverse the handedness of circularly polarized (CP) waves, i.e., to convert between right-circularly polarized (RCP) and left-circularly polarized (LCP) waves. This functionality not only plays a key role in metasurface devices based on the Pancharatnam-Berry (PB) phase [1, 2] but also provides the conditions for achieving full-Stokes polarization control [3]. Accordingly, the co- and cross-polarization transmittances for CP waves incident in the forward and backward directions at room temperature are calculated according to equations (4a)-(4d) in the main text, and the results are presented in Figure S2(a) and (b), respectively. The results show that over a wide frequency range of 3.59 THz - 5.65 THz, the proposed TMPMM can convert RCP/LCP waves with different incident directions into LCP/RCP waves, with polarization conversion transmittance exceeding 0.8. At 4.85 THz, T^f_{-+} and T^b_{+-} reach a maximum of 0.982, while T^f_{+-} and T^b_{-+} reach a maximum of 0.978, achieving near-perfect cross-polarization conversion for CP waves.

In contrast, metasurface structures currently used for multifunctional polarization control primarily employ ultrathin metal resonators as polarization conversion elements [4–16]. However, these designs are constrained by the theoretical limits of single-layer ultrathin metallic structures in transmission mode: the conversion efficiency from LP to its orthogonal polarization typically does not exceed 0.25 [17], and that from LP to CP is generally below 0.5 [18]. To overcome these limitations, existing designs generally enhance polarization conversion performance by increasing the number of metal layers and utilizing multiple reflections between the front and back surfaces to form Fabry-Pérot-like cavities. Depending on the structural integration method, current research is primarily advancing in several directions:

Metallic resonators are integrated with two mutually orthogonal double-layer metal gratings placed on opposite sides [4–8]. This configuration exploits the dual polarization-selective properties of the orthogonal gratings, typically enabling efficient cross-polarization conversion over a broad bandwidth and facilitating asymmetric transmission (AT). However, because the front and back gratings constrain the polarization states at the input and output, respectively, such structures, when functioning as a polarization device,

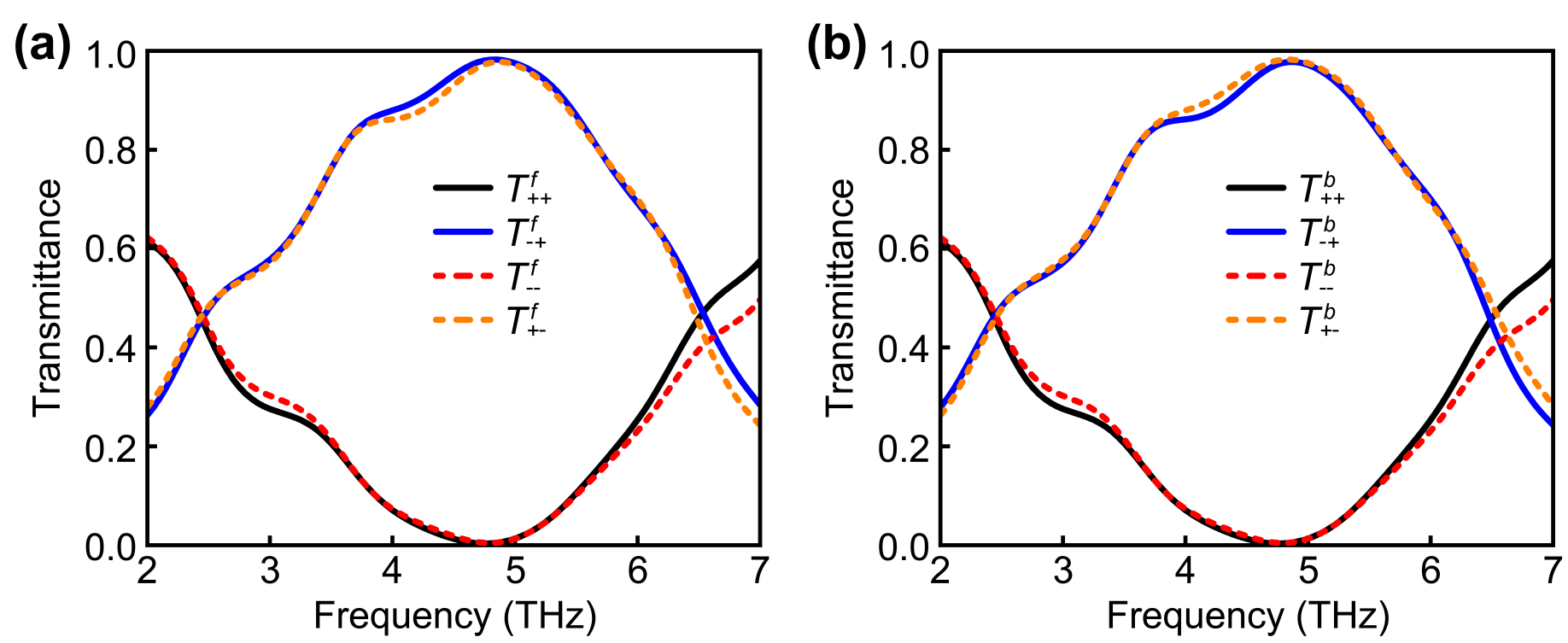

Figure S2. Transmission spectra of co- and cross-polarized for (a) forward- and (b) backward-incident RCP and LCP waves at room temperature, respectively.

generally operate efficiently only for LP waves whose direction of polarization is perpendicular to the front grating. As a result, it cannot effectively modulate CP waves nor directly output CP waves.

Metallic resonators are integrated with a single-layer metallic grating on one side [9–12], typically with the input grating removed. This configuration enables polarization conversion for forward-incident LP and CP waves. However, since the output port remains constrained by the polarization-selective properties of the metallic grating, the structure can only output an LP wave in a single direction and requires the incident LP waves with a specific direction or CP waves with a specific handedness. Consequently, in transmission mode, the structure cannot fully function as an HWP or quarter-wave plate (QWP) for incident waves with different polarization states. Moreover, since the conversion efficiency from linear to orthogonal polarization is lower than that from linear to circular polarization [17, 18], this configuration generally suffers from low transmittance when achieving cross-polarization conversion for LP wave in a specific direction [11, 19], compared to schemes designed for circular polarization conversion [9]. To address these limitations, some studies have proposed integrating VO_2 grating around the metallic grating, utilizing high temperatures to transform VO_2 into the metallic state, thereby effectively achieving waveplate functionality in the reflection mode [10, 12]. However, reflective polarization control faces challenges in practical applications, such as feed blockage and the need for additional beam-steering components, making it difficult to integrate into optical paths as conveniently as transmissive devices [20–23]. Furthermore, to obtain CP wave output in transmission, a multilayer structure consisting of “metallic resonator-metallic grating-metallic resonator” has been proposed [10, 24], using the bottom metallic resonator to convert the LP wave transmitted by the grating into a CP wave. Nevertheless, since the metal grating can only transmit an LP wave in a single direction, the handedness of the resulting output CP wave is also fixed, lacking tunability.

Metallic resonators are integrated with their flipped or rotated structures [13–16]. Owing to the lack of strong polarization-selective properties of metallic gratings, such metasurfaces typically offer greater flexibility regarding the polarization states of both incident and transmitted waves when achieving polarization conversion. However, their performance is often limited, exhibiting lower polarization conversion transmittance, weaker asymmetric transmission (AT) effects, or narrower operational bandwidths compared to the first two types of structures, especially the first type. To address this limitation, VO_2 material is integrated around the bottom metallic resonators [14], enabling a switch to reflective mode at high temperatures, thereby achieving HWP functionality while improving polarization conversion performance.

In summary, the currently proposed multifunctional polarization conversion metasurfaces typically suffer from a trade-off between performance and multifunctionality. Moreover, due to limitations imposed by AT characteristics, complete HWP or QWP functionality often needs to be realized in reflection mode. In contrast, the performance of the TMPMM proposed in this work is essentially free from the constraints of grating polarization selectivity at room temperature. It can perform the corresponding waveplate functions for incident waves of different polarization states in transmission, with polarization conversion transmittance approaching unity. This provides a foundation for realizing efficient, multifunctional, and tunable polarization manipulation in transmission.

3 Supplementary Note 3 Analysis of backward-incident x-polarized transmission via Jones matrix under room temperature

As analyzed in Section 3.1 of the main text, for backward-incident x- and y-polarized waves, the cross-polarized transmission coefficients are reversed compared to forward-incidence ($t^f_{yx} = t^b_{xy}$ and $t^f_{xy} = t^b_{yx}$), whereas the co-polarized coefficients remain unchanged ($t^f_{xx} = t^b_{xx}$ and $t^f_{yy} = t^b_{yy}$). Therefore, the transmitted electric field under backward incidence can be expressed as

$$\begin{pmatrix} E_x^{t(b)} \\ E_y^{t(b)} \end{pmatrix} = \frac{1}{2} \begin{bmatrix} (t_{uu} - t_{uv}) - (t_{vu} - t_{vv}) & (t_{uu} - t_{uv}) + (t_{vu} - t_{vv}) \\ (t_{uu} + t_{uv}) - (t_{vu} + t_{vv}) & (t_{uu} + t_{uv}) + (t_{vu} + t_{vv}) \end{bmatrix} \begin{pmatrix} E_x^{i(b)} \\ E_y^{i(b)} \end{pmatrix}$$

$$= \begin{bmatrix} t_{xx}^b & t_{xy}^b \\ t_{yx}^b & t_{yy}^b \end{bmatrix} \begin{pmatrix} E_x^{i(b)} \\ E_y^{i(b)} \end{pmatrix} \tag{S1}$$

Taking x-polarized incidence as an example for detailed analysis, substituting into $[1 \quad 0]^T$ yields the transmitted electric field as:

$$\begin{pmatrix} E_x^{t(b)} \\ E_y^{t(b)} \end{pmatrix} = \begin{pmatrix} t_{xx}^b \\ t_{yx}^b \end{pmatrix} = \frac{1}{2} e^{\mathrm{i}\Phi_{vv}} \begin{pmatrix} a_{uu}e^{\mathrm{i}\Delta\Phi_1} + a_{vv} - a_{vu}e^{\mathrm{i}\Delta\Phi_2} - a_{uv}e^{\mathrm{i}\Delta\Phi_3} \\ a_{uu}e^{\mathrm{i}\Delta\Phi_1} + a_{uv}e^{\mathrm{i}\Delta\Phi_3} - a_{vu}e^{\mathrm{i}\Delta\Phi_2} - a_{vv} \end{pmatrix} \tag{S2}$$

Near 4.8 THz, based on $\Delta\Phi_2 \approx 1.5\pi$, $\Delta\Phi_3 \approx 0.5\pi$, and $a_{vu} \approx a_{uv}$, we obtain:

$$\begin{pmatrix} E_x^{t(b)} \\ E_y^{t(b)} \end{pmatrix} = \frac{1}{2} e^{\mathrm{i}\Phi_{vv}} \begin{pmatrix} a_{uu}e^{\mathrm{i}\Delta\Phi_1} + a_{vv} \\ a_{uu}e^{\mathrm{i}\Delta\Phi_1} + 2a_{uv}e^{\mathrm{i}\Delta\Phi_3} - a_{vv} \end{pmatrix} \tag{S3}$$

For t_{xx}^b, because $a_{uu} \approx a_{vv} \approx 1$ and $\Delta\Phi_1 \approx \pi$, $\mid t_{xx}^b \mid \approx 0$. For t_{yx}^b, since $a_{uv} \approx 0.064 \ll 1$, $\mid t_{yx}^b \mid \approx \mid a_{uu} \mid \approx 1$, thereby achieving a nearly ideal orthogonal polarization conversion.

In the low-frequency region around 2.45 THz, both a_{vu} and a_{uv} remain below 0.035 and are negligible compared with a_{uu} and a_{vv}. The transmitted field thus simplifies to:

$$\begin{pmatrix} E_x^{t(b)} \\ E_y^{t(b)} \end{pmatrix} = \frac{1}{2} e^{\mathrm{i}\Phi_{vv}} \begin{pmatrix} a_{uu}e^{\mathrm{i}\Delta\Phi_1} + a_{vv} \\ a_{uu}e^{\mathrm{i}\Delta\Phi_1} - a_{vv} \end{pmatrix} \tag{S4}$$

Here, a_{uu} and a_{vv} exhibit high and comparable magnitudes, and $\Delta\Phi_1 \approx 1.5\pi$. Therefore, the conversion from linearly polarized (LP) to right-circularly polarized (RCP) waves is effectively achieved.

In the high-frequency region around 6.55 THz, substituting $a_{vu} = 0.109$, $a_{uv} = 0.094$, $a_{uu} = 0.911$, $a_{vv} = 0.957$, $\Delta\Phi_1 = 0.506\pi$, $\Delta\Phi_2 = 0.929\pi$, and $\Delta\Phi_3 = 0.382\pi$ into equation (S2) gives

$$\begin{pmatrix} E_x^{t(b)} \\ E_y^{t(b)} \end{pmatrix} = \frac{1}{2} e^{\mathrm{i}\Phi_{vv}+0.213\pi} \begin{bmatrix} 1.29 \\ 1.28e^{\mathrm{i}0.513\pi} \end{bmatrix} \tag{S5}$$

It can be observed that the amplitudes of $E_x^{t(b)}$ and $E_y^{t(b)}$ are close to each other, and their phase difference is approximately $\pi/2$, thereby effectively achieving the conversion from LP to left-circularly polarized (LCP) waves.

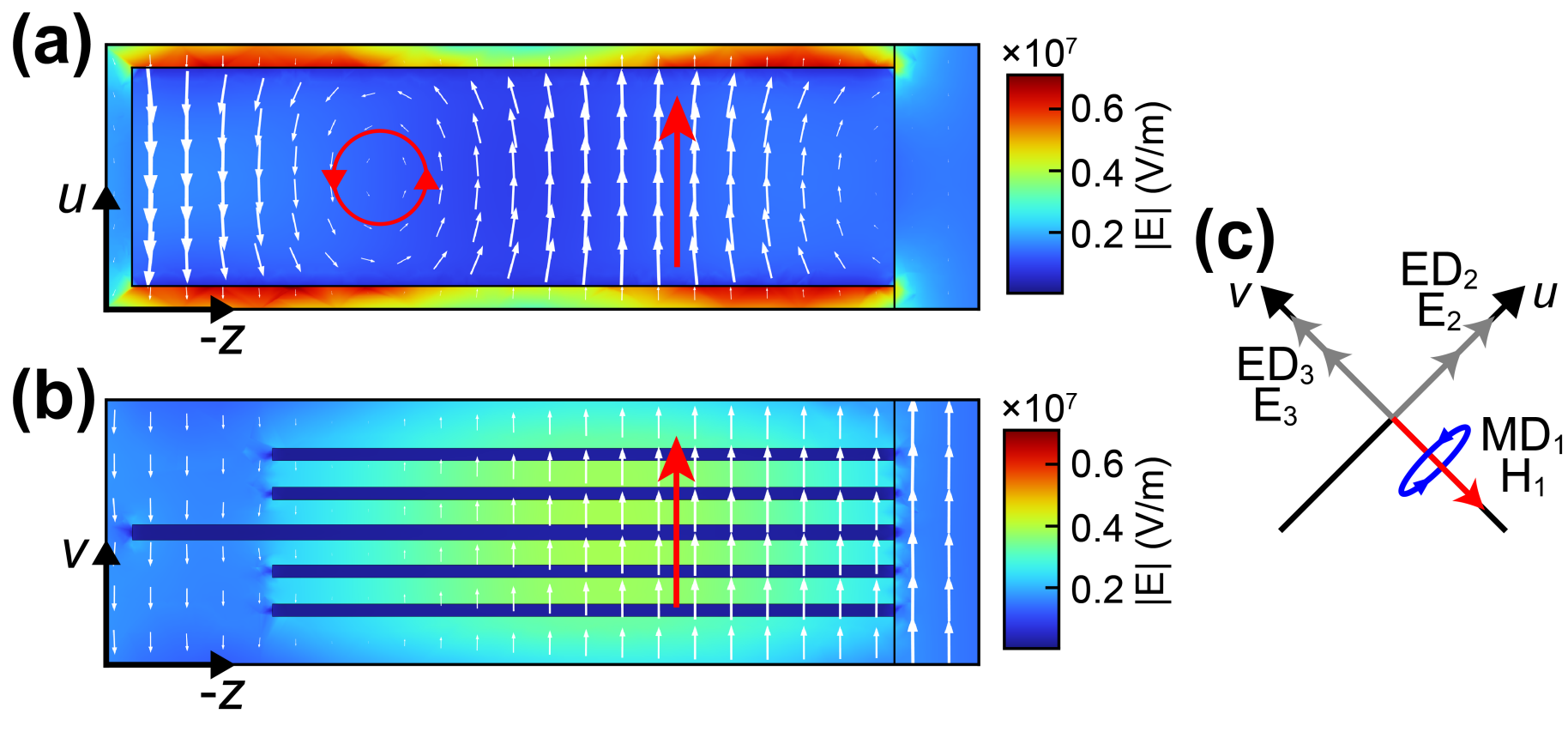

Figure S3. Electric field distribution at 2.45 THz. The color bars represent the field magnitude, and white arrows represent current vectors. (a) Electric field distribution on the u-z plane with u-polarized incidence. (b) Electric field distribution on the v-z plane with v-polarized incidence. (c) MD_1, ED_2, and ED_3.

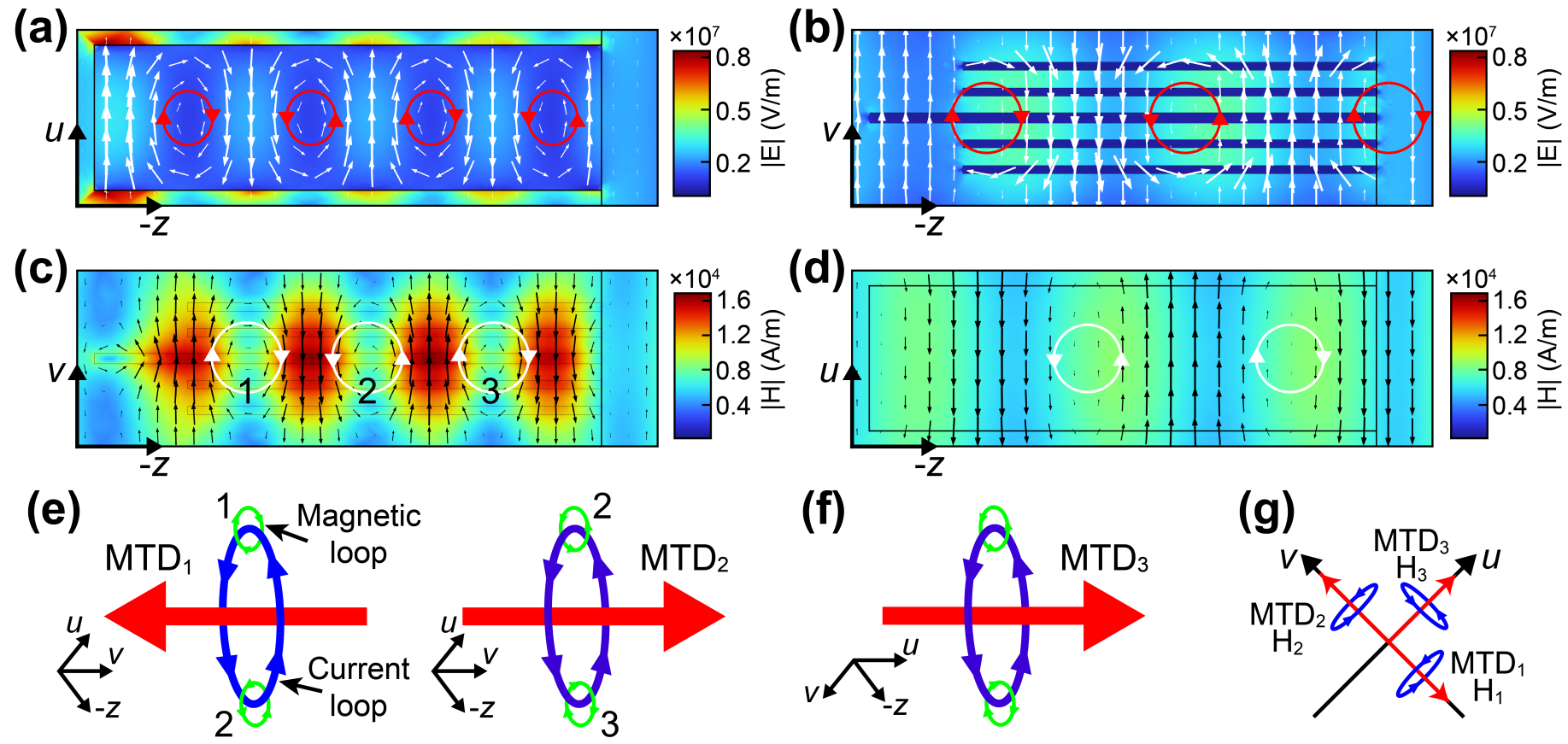

Figure S4. Electromagnetic field distributions at 6.55 THz. The color bars represent field magnitude. White and black arrows represent current vectors and magnetic vectors, respectively. (a) Electric field distribution in the u-z plane and (c) magnetic field distribution in the v-z plane with u-polarized incidence. (b) Electric field distribution in the v-z plane and (d) magnetic field distribution in the u-z plane with v-polarized incidence. (e) and (f) Schematic diagrams of MTD formation. (g) MTD_1, MTD_2, and MTD_3.

4 Supplementary Note 4 Analysis of the physical mechanism of linear-to-circular polarization conversion

At a frequency of 2.45 THz, the electric field distributions with u- and v-polarized incident are shown in Figure S3(a) and (b), respectively, where the white arrows represent current vectors. For u-polarized incidence, a counterclockwise ring current is observed on the u-z plane near the top of the dielectric pillars, exciting a magnetic dipole (MD_1) mode along the v-axis, as marked by the red circle in Figure S3(a). Additionally, due to the non-uniform current distribution along the height of the pillars (i.e., the z-direction), an electric dipole (ED_2) along the u-axis is also excited at its mid-lower region, as indicated by the red arrow in Figure S3(a). For v-polarized incidence, the electric field is primarily localized in the gaps between adjacent dielectric pillars, and the current direction is consistently along the positive v-axis. This indicates the excitation of an ED_3 mode along the v-axis. As illustrated in Figure S3(c), the transmission amplitude and phase of the u-polarized wave are jointly modulated by MD_1 and ED_2, while those of the v-polarized wave are governed by ED_3. The resulting amplitudes are comparable, and the phase difference is approximately 90°, thereby efficiently achieving conversion from LP to circularly polarized (CP) waves.

At a frequency of 6.55 THz, the electric- and magnetic-field distributions with u-polarized incidence are shown in Figure S4(a) and (c), respectively. Those with v-polarized incidence are presented in Figure S4(b) and (d), respectively. In the figures, white arrows denote current vectors, and black arrows represent magnetic-field vectors. For u-polarized incidence, the field distributions reveal that four current loops form in the u-z plane, with adjacent loops rotating in opposite directions, as marked by the red circles in Figure S4(a). This indicates the excitation of four MD modes along the v-axis. Further inspection of the magnetic field distribution in Figure S4(c) reveals that adjacent MDs are coupled to form magnetic loops. Thus, the four MD modes in Figure S4(a) collectively generate three magnetic loops, indicated by white circles in Figure S4(c). For easy discussion, these loops are labeled as 1, 2, and 3. Magnetic loops 1 and 2 couple to excite an MTD_1 mode along the $-v$-direction, while loops 2 and 3 couple to produce an MTD_2 mode along the $+v$-direction, as shown in Figure S4(e). For v-polarized incidence, three current loops are observed on the v-z plane, as indicated by the red circles in Figure S4(b). The three MD modes excite two counter-rotating magnetic loops on the u-z plane, generating the MTD_3 mode along the u-axis, shown in Figure S4(f). Consequently, the amplitude and phase of the u-polarized wave are jointly modulated by MTD_1 and MTD_2, while those of the v-polarized wave are mainly governed by MTD_3, as summarized in Figure S4(g). Because the amplitudes of the two polarized components differ slightly and their phase deviation slightly exceeds 90°, conversion from LP to CP waves is still achievable, yet the resulting ellipticity

$|\eta|$ is limited to below 0.9.

5 Supplementary Note 5 Analysis of *y*-polarized transmission via Jones matrix under higher temperatures

The transmitted electric field for forward- and backward-incident y-polarized waves can be approximated as

$$\begin{pmatrix} E_x^{t(f)} \\ E_y^{t(f)} \end{pmatrix} = \frac{1}{2} e^{\mathrm{i}\Phi_{vv}} \begin{bmatrix} (a_{uu} - a_{vu})(e^{\mathrm{i}\Delta\Phi_1} - 1) \\ e^{\mathrm{i}\Delta\Phi_1}(a_{uu} + a_{vu}) + (a_{uv} + a_{vv}) \end{bmatrix} \tag{S6}$$

$$\begin{pmatrix} E_x^{t(b)} \\ E_y^{t(b)} \end{pmatrix} = \frac{1}{2} e^{\mathrm{i}\Phi_{vv}} \begin{bmatrix} e^{\mathrm{i}\Delta\Phi_1}(a_{uu} + a_{vu}) - (a_{uv} + a_{vv}) \\ e^{\mathrm{i}\Delta\Phi_1}(a_{uu} + a_{vu}) + (a_{uv} + a_{vv}) \end{bmatrix} \tag{S7}$$

From equations (S6) and (S7), it follows that $| t_{xy}^f |$ remains close to zero across the broad frequency band of 2-7 THz because the term $(a_{uu} - a_{vu})$ is relatively weak and $| t_{yy}^f |$ and $| t_{yy}^b |$ approach zero when $\Delta\Phi_1 = \pi$; $| t_{xy}^b |$ reaches near-unity at $\Delta\Phi_1 = \pi$. Therefore, the proposed tunable multifunctional polarization manipulating metasurface (TMPMM) exhibits an AT effect close to -1 for the y-polarized wave around 4.95 THz. At other frequencies, since $e^{\mathrm{i}\Delta\Phi_1} \neq -1$, both t_{yy}^f and t_{yy}^b will have certain components. However, because their amplitudes are equal ($| t_{yy}^f | = | t_{yy}^b |$), their contributions cancel each other in the calculation of the asymmetric transmission (AT) parameter, thus not affecting the overall AT effect. As a result, for both x-polarized and y-polarized waves incident, the AT effect of the structure is primarily determined by the term $e^{\mathrm{i}\Delta\Phi_1}(a_{uu} + a_{vu}) - (a_{uv} + a_{vv})$. While the term $(a_{uu} - a_{vu})\,(e^{\mathrm{i}\Delta\Phi_1} - 1)$ can generally be neglected due to its weak magnitude.

6 Supplementary Note 6 Electric-field distributions for asymmetric transmission

Figure S5(a) and (b) show the electric field distributions at 5 THz for x- and y-polarized waves with forward and backward illumination, respectively. It is clearly observed that the forward-incident x-polarized wave, after being converted by the Ge dielectric pillars, effectively couples with the VO_2 grating, exciting strong field distributions and thereby achieving high transmission efficiency. In contrast, the backward-incident x-polarized wave is reflected due to its failure to efficiently excite surface plasmon polaritons [25]. The weak field distribution in the top layer further confirms that the backward-incident x-polarized wave is reflected into the original medium. For y-polarized incidence, the transmission and reflection situations are reversed. The field maps show that the backward-incident y-polarized wave couples effectively with the VO_2 grating, leading to high transmission efficiency, while the forward-incident y-polarized wave is efficiently reflected after being converted into an x-polarized wave by the Ge dielectric pillars.

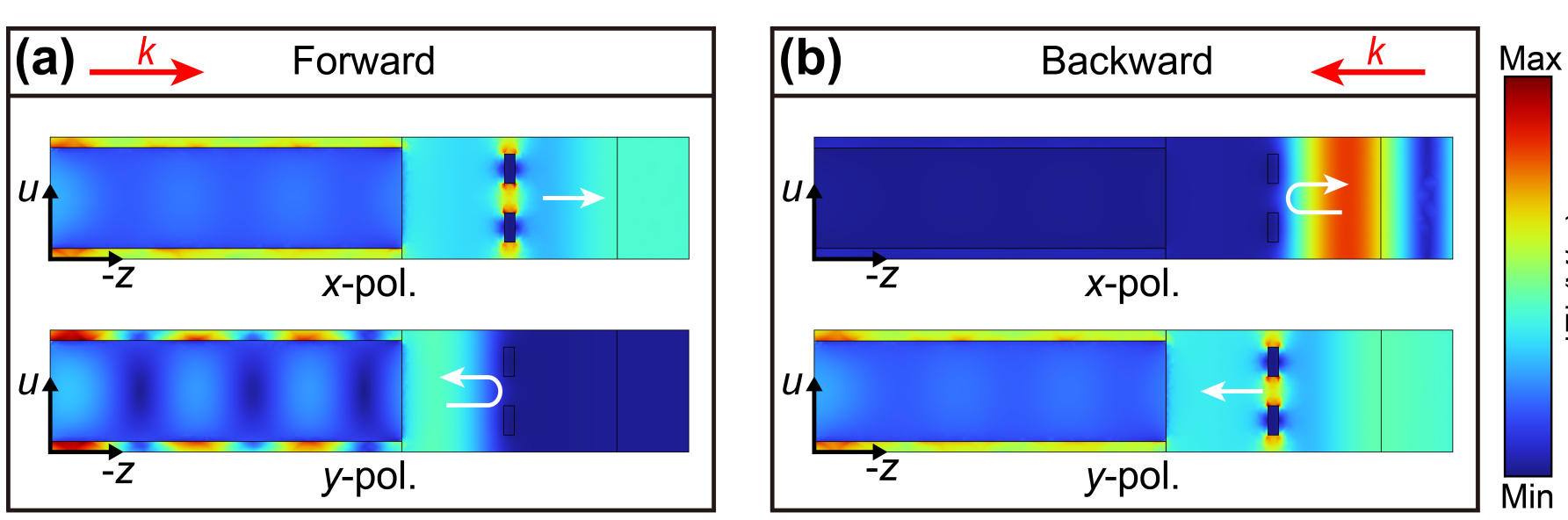

Figure S5. Electric field distributions in the u-z plane for x- and y-polarized waves with (a) forward- and (b) backward-illumination.

7 Supplementary Note 7
Polarization conversion ratio of the CP and LP waves under various polarization angles

To verify that TMPMM can convert LP waves into RCP and y-polarized waves regardless of the polarization angle, the polarization conversion ratios (PCRs) for RCP and y-polarized waves are calculated under various excitation angles. The PCR for the RCP wave is defined as

$$PCR_{+} = \frac{R_{+}^{f}}{R_{+}^{f} + R_{-}^{f}} \tag{S8}$$

Here, the superscript f denotes forward propagation of the LP wave along the $-z$ direction, while the subscripts $+$ and $-$ correspond to RCP and LCP waves, respectively. The polarization angle θ of the incident LP wave is defined as the angle between the incident electric field and the x-axis, as illustrated in Figure S6(a). Figure S6(b) and (c) show the variation trends of PCR_{+} and PCR_{y} as a function of θ continuously varying from 0° to 180°, across the frequency ranges of 2.24 THz - 2.28 THz and 2 THz - 2.6 THz, respectively. The results reveal that PCR_{+} exhibits a sinusoidal-like trend with increasing θ, where its amplitude initially increases, then decreases, and subsequently increases again. Notably, PCR_{+} remains above 0.91 for all polarization angles, reaching a maximum value of 0.987. This demonstrates that the proposed TMPMM exhibits low-polarization-sensitivity linear-to-circular polarization conversion characteristics around 2.25 THz. On the other hand, over the entire frequency range of 2 THz - 2.6 THz, PCR_{y} remains above 0.99, indicating that LP waves incident at any polarization angle are nearly perfectly converted into y-polarized waves. Leveraging these unique properties, the system can significantly reduce the requirement for polarization calibration in practical applications, thereby offering enhanced flexibility and applicability for practical scenarios such as wireless communications, imaging, and optical waveplates [26–28].

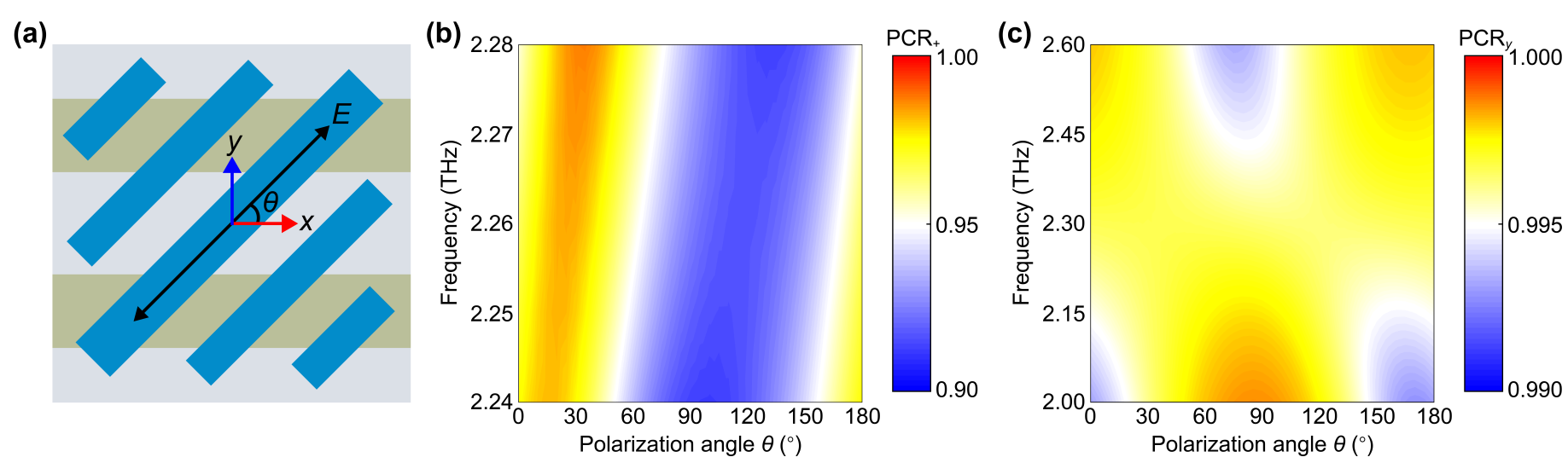

Figure S6. (a) Definition of the incident wave polarization angle θ. Spectra of (b) PCR_{+} and (c) PCR_{y} under various excitation angles.

8 Supplementary Note 8
Broadband strong chiral response and robust polarization conversion via structural optimization of Ge dielectric pillars and grating

By optimizing the geometric parameters of the Ge dielectric pillars and replacing the grating material with silver, the proposed metasurface structure simultaneously achieves strong chiral responses ($|\mathrm{CD}| > 0.8$) with a bandwidth exceeding 2.75 THz in both transmission and reflection; as shown in Figure S7. The adjusted parameters of the Ge dielectric pillars are as follows: $h_1 = 27$ µm, $h_2 = h_3 = 20$ µm, $a_2 = a_3 = 0.7$ µm, $g_1 = 1.65$ µm, and $g_2 = 1.8$ µm, while all other parameters remain consistent with those in Figure 1 of the main text. The relative permittivity of silver was calculated using the Drude model [29]. Figure S7(a) and (b) show the transmission and reflection spectra for forward-incident RCP and LCP waves, respectively. It can be seen that over a broad frequency range of 3.7 THz - 5.7 THz, for incident RCP wave, the reflectance of the co-polarized wave is consistently greater than 0.928, reaching 0.982 and 0.989 at 3.87 THz and 5.45

THz, respectively, while the cross-polarized reflectance, co-polarized transmittance, and cross-polarized transmittance all remain below 0.029. This indicates that the incident RCP wave is efficiently reflected while preserving its helicity. For incident LCP wave, both the co- and cross-polarized reflectance remain below 0.045 over this frequency band, whereas the co- and cross-polarized transmittances are nearly equal and both exceed 0.473. This indicates that the incident LCP wave efficiently passes through the metasurface structure and is converted into an LP wave, as illustrated by the orange curve in Figure S7(c). Thus, the proposed structure selectively reflects RCP wave and transmits LCP wave over the broad bandwidth of 3.7 THz - 5.7 THz. Figure S7(d) and (e) show the CD spectra in reflection and transmission, respectively. The simulation results demonstrate that within the broad bandwidth of 3.37 THz - 6.14 THz, CD_R remains below -0.8, reaching as low as -0.964 and -0.963 at 3.85 THz and 5.38 THz, respectively. Over a broad bandwidth of 3.35 THz - 6.14 THz, CD_T remains above 0.8, reaching as high as 0.969 and 0.967 at 3.85 THz and 5.38 THz, respectively.

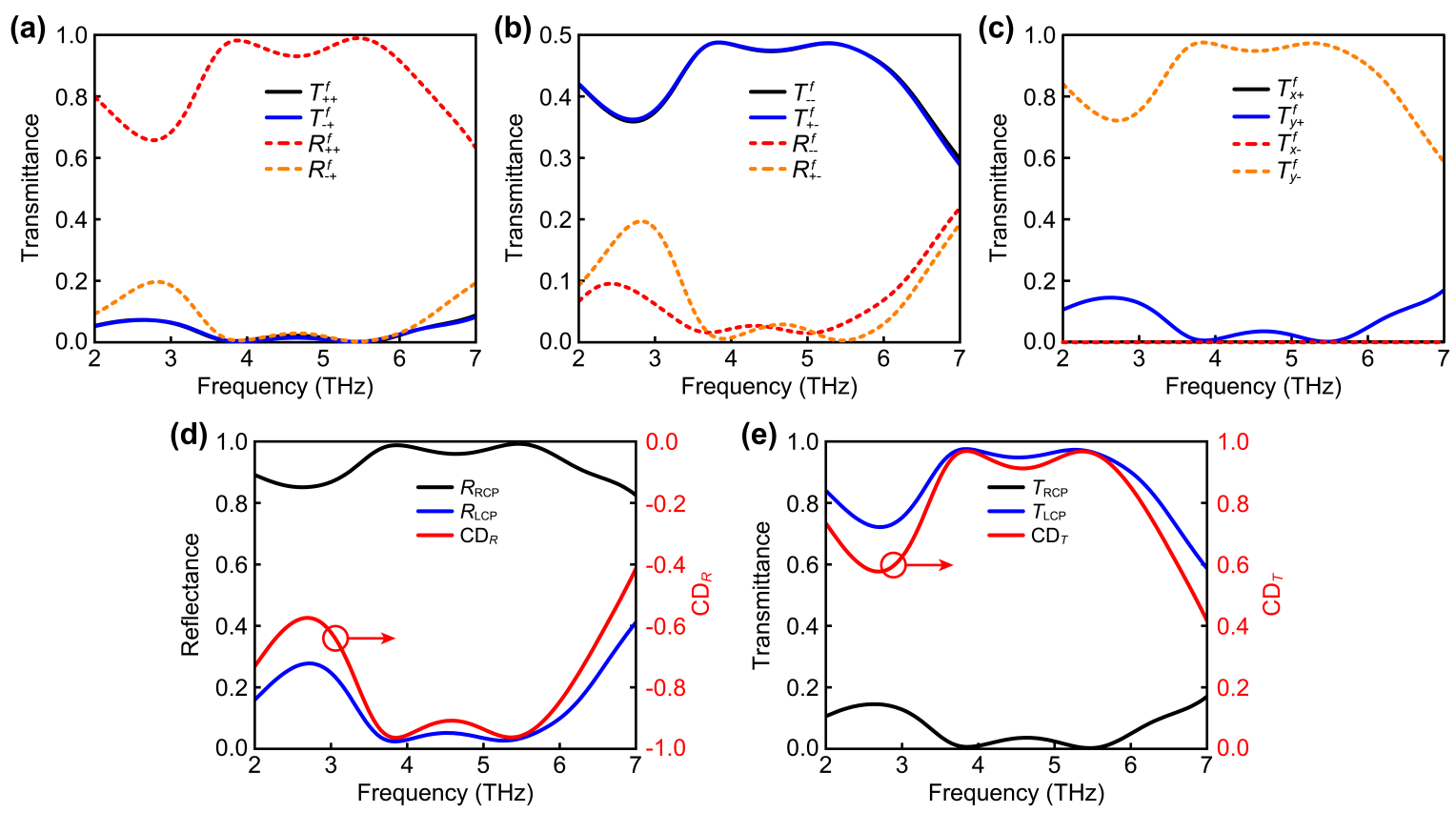

Figure S7. Spin-dependent polarization selection results after structural optimization. Co- and cross-polarized transmittance and reflectance spectra after forward-incident (a) RCP and (b) LCP waves illumination of the TMPMM. (c) LP components in transmission. (d) Total reflectance under forward-incident RCP (black curve) and LCP (blue curve) waves, and their reflectance difference (red curve). (e) Total transmittance under forward-incident RCP (black curve) and LCP (blue curve) waves, and their transmittance difference (red curve).

Owing to the significant improvement in chiral response, the optimized metasurface structure also exhibits marked performance enhancement in achieving robust CP conversion and near-perfect LP output, as shown in Figure S8. Figure S8(a) and (b) illustrate the variation trends of PCR_+ and PCR_y over a broad frequency range as θ continuously varies from 0° to 180°. The results indicate that within the broad frequency band of 3.7 THz - 5.7 THz, PCR_+ remains above 0.91 for all polarization angles, achieving broadband, low-polarization-sensitive polarization conversion from LP to CP waves. In the 3 THz - 6 THz band, PCR_y remains above 0.998 for all polarization angles, achieving broadband near-perfect LP output. In summary, leveraging the efficient polarization conversion capability of the Ge dielectric pillars and the polarization-selective characteristics of the grating, the proposed metasurface provides a crucial platform for developing high-performance terahertz chiral response devices.

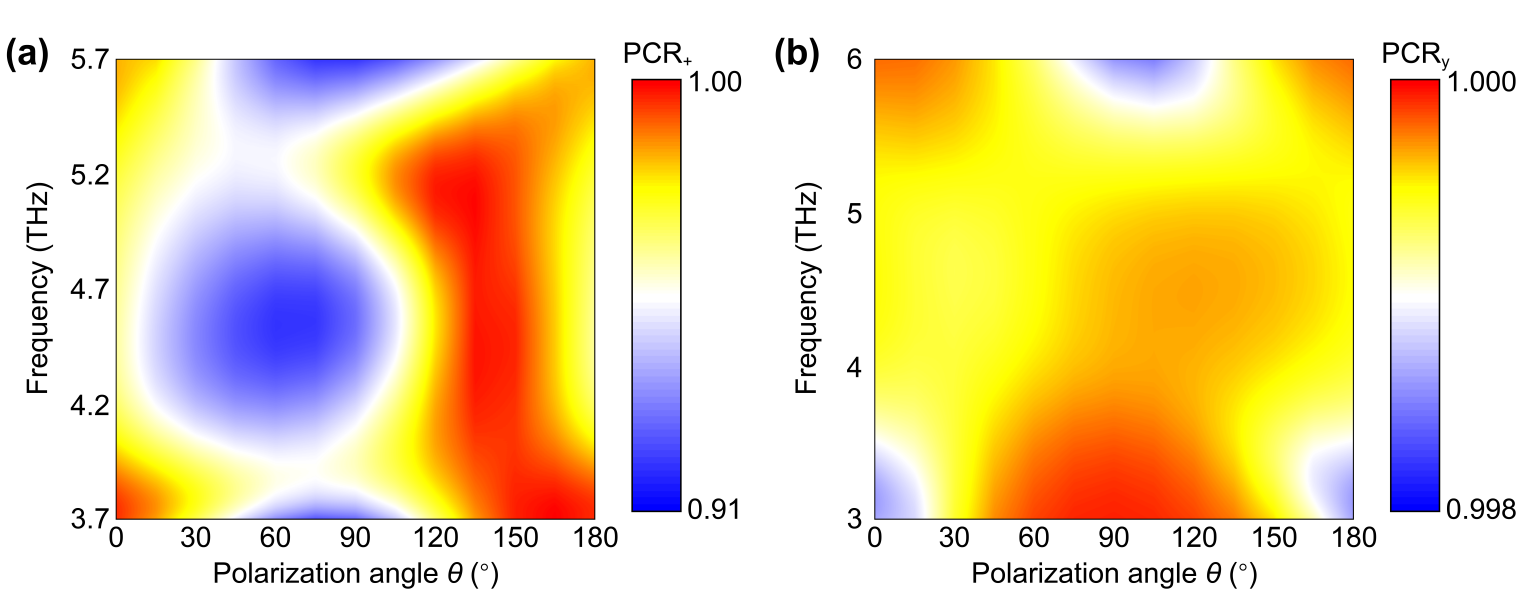

Figure S8. Spectra of (a) PCR_+ and (b) PCR_y under various excitation angles.

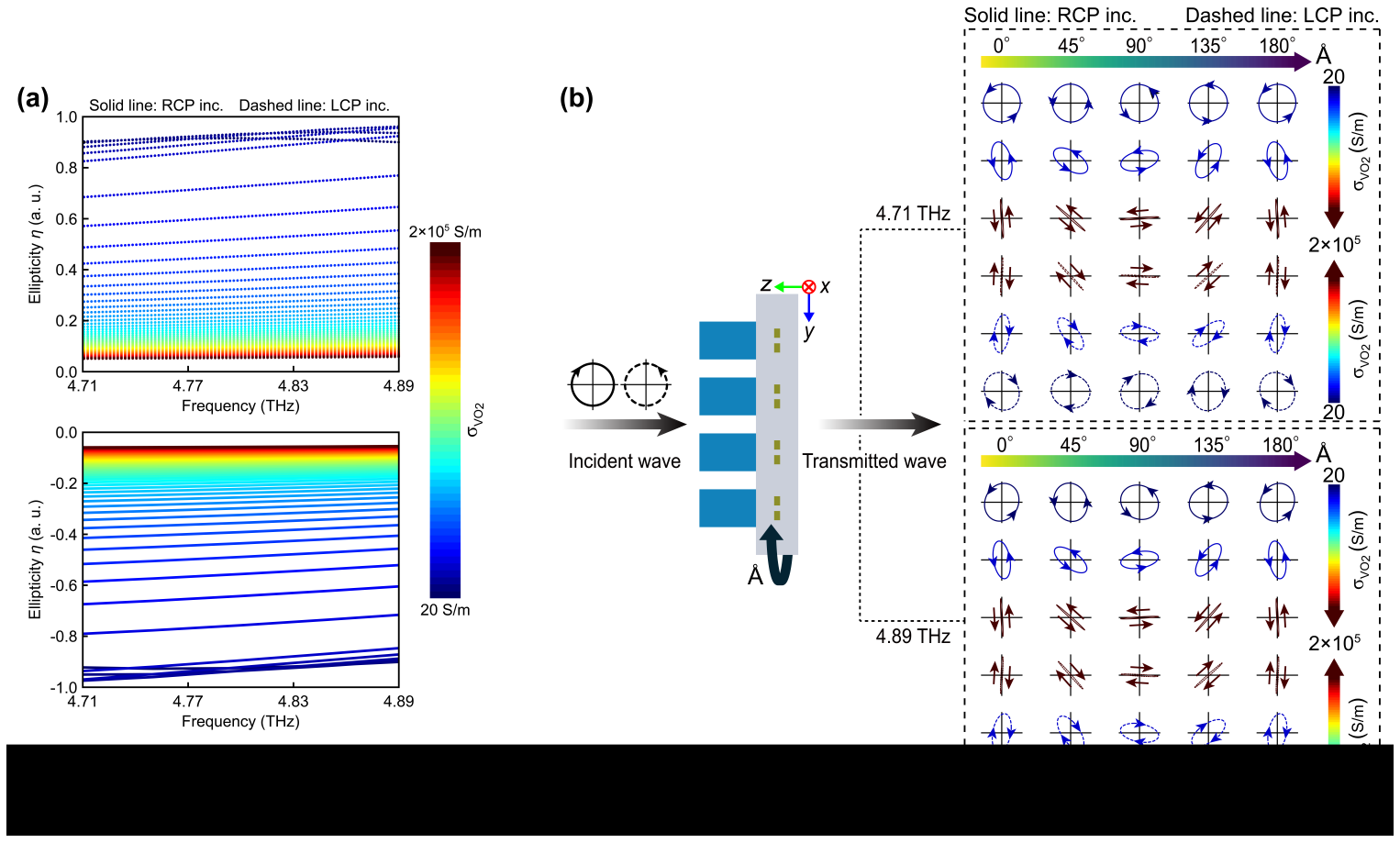

Figure S9. (a) Ellipticity distributions for forward-incident RCP (solid lines) and LCP (dashed lines) waves across 4.71 THz - 4.89 THz under different σ_{VO_2}. (b) Schematic illustration of full-Stokes polarization control through geometric polarization and dynamic tuning of σ_{VO_2} at 4.71 THz and 4.89 THz.

9 Supplementary Note 9 Broadband full-Stokes polarization control

Figure S9 (a) shows the ellipticity response of the transmitted waves after RCP and LCP waves incident on the TMPMM under different σ_{VO_2}, where the solid and dashed lines represent RCP and LCP incident, respectively, and the color shading indicates the magnitude of σ_{VO_2}. The results show that over a broad frequency range of 4.71 THz - 4.89 THz, as σ_{VO_2} gradually increases from 20 S/m to 2×10^5 S/m, $|\eta|$ under forward-incident RCP/LCP wave can be effectively tuned from above 0.9 to below 0.1. Unlike the case of LP wave incidence discussed in Section 3.4.3 of the main text, CP wave incidence can generate unique geometric phases when the structure rotates [3], with a distinctive characteristic: while keeping η of the transmitted wave unchanged, polarization azimuth angle satisfies the relationship $\varphi' = \varphi + \text{Å}$ as the rotation angle Å changes, where φ and φ' represent the polarization azimuth angles before and after rotation, respectively. Consequently, when Å varies from 0° to 180°, the polarization ellipse of the transmitted wave rotates synchronously by 180°, coinciding with that in the unrotated case, thereby achieving full coverage of the polarization azimuth angle, as illustrated in each row of Figure S9(b). Furthermore, by adjusting σ_{VO_2}, the ellipticity can be tuned independently and continuously at different frequencies. Combined with the linear modulation of the azimuth angle, the structure enables full-Stokes polarization control at any frequency within the 4.71 THz - 4.89 THz range. The evolution trend can be referenced from the representative polarization ellipse evolutions selected at 4.71 THz and 4.89 THz, as shown in Figure S9(b). Benefiting from the efficient dynamic tunability of the TMPMM, full-Stokes polarization control can be achieved without relying on frequency multiplexing or integration of multiple structures. This means that polarization control, which was originally limited to motion along latitude circles on the Poincaré sphere at a single frequency, has been extended to the entire sphere, representing a significant breakthrough in enhancing the dimensionality and information capacity of polarization control.

10 Supplementary Note 10 Linear tuning of the operating frequency band via overall scaling of the TMPMM

By uniformly scaling the geometrical dimensions of the structure, the operating frequency of the proposed TMPMM can be effectively extended to other frequency bands, as shown in Figure S10. To systematically investigate this scalability, we first conducted detailed analyses of the metasurface structure without the VO_2 grating and of the structure without Ge dielectric pillars, respectively. A scaling factor S is used to

indicate the scaling ratio of all parameters of the Ge dielectric pillars and substrate with regard to the original one proposed in the manuscript. The simulation results of co/cross polarization transmittance are shown in Figure S10(a)-(d).

Figure S10(a) and (b) illustrate the co/cross polarization transmittances for an x-polarized wave incident on the metasurface structure (without VO_2 grating) for $S = 3000$ and $S = 1/10$, respectively. Considering that SiO_2 is rarely used as a substrate material for metasurfaces in the GHz band, a common polytetrafluoroethylene (PTFE) is selected as the substrate with a permittivity of 2.1, which is comparable to the refractive index of the SiO_2, and exhibits negligible dielectric loss [30,31]. As shown in Figure S10(a), when $S = 3000$, the metasurface without a VO_2 grating can efficiently convert an incident x-polarized wave into a y-polarized wave ($T_{yx} > 0.8$) over the frequency range of 1.22 GHz - 1.92 GHz, with a relative bandwidth of 44.6%. As shown in Figure S10(b), when $S = 1/10$, the same polarization conversion functionality is achieved over the range of 35.81 THz - 56.87 THz, with a relative bandwidth of 45.4%. Furthermore, Figure S1(c) in the Supplementary Note 1 indicates that for the original case of $S = 1$, the structure efficiently converts x-polarized wave to y-polarized wave over the frequency range of 3.59 THz - 5.69 THz, with a relative bandwidth of 45.3%. Accordingly, the designed Ge-dielectric-pillar structure can stably achieve high-efficiency half-wave plate functionality with a relative bandwidth of approximately 45% across scaling factors ranging from $S = 1/10$ to $S = 3000$.

For the structure containing only VO_2 grating (without Ge pillars), the co-polarization transmittances for both x- and y-polarized incident waves with $S = 3000$ and $S = 1/4$ are shown in Figure S10(c) and (d), respectively. The conductivity of VO_2 was selected to be 1 S/m (Figure S10(c)) and 2×10^5 S/m (Figure S10(d)), respectively [32]. Simulation results of co-polarization transmittance indicate that when $S = 3000$, the VO_2 grating in the insulating state cannot effectively transmit x- and y-polarized waves, particularly for x-polarized wave (as shown by the black curve in Figure S10(c)). According to the Drude model of VO_2, when the conductivity σ_{VO_2} is not exactly zero (nonideal case), the significant reduction in the frequency of the incident wave introduces impedance mismatch and enhanced loss, causing the low transmittance of both polarizations. When $S = 1/4$, the VO_2 grating in the metallic state cannot effectively suppress the transmission of x-polarized wave (as shown by the black curve in Figure S10(d)).As indicated in the Drude model of VO_2, limited by the plasma frequency $\omega_p(\sigma_0)$ of VO_2, which is only 1.4×10^{15} rad/s. When the conductivity increases to 2×10^5 S/m, its plasma frequency is far lower than that of noble metals such as gold or silver, limiting its performance as a polarizer in the higher THz frequency band. In summary, the expandable range of operating frequencies depends on the applicable regions where VO_2 achieves high-efficiency transmission in the insulating state and effective selective transmission in the metallic state, respectively.

By systematically performing numerical simulations with various scaling factors S, we find that the proposed TMPMM operates effectively over a scaling range from $S = 2/3$ to $S = 300$. The corresponding performances of functioning as a half-wave plate, quarter-wave plate, and asymmetric transmission are shown in Figure S10(e)-(p), respectively. For easy comparison, the performance of the TMPMM with $S = 3000$ and $S = 1/4$ are shown in Figure S10(q) and (r), respectively. The results show that when $S = 3000$ and $\sigma_{VO_2} = 1$ S/m, the maximum transmittance of cross-polarization conversion T^f_{xy} for an incident y-polarized wave is 0.615 (as shown by the orange curve in Figure S10(q)), indicating that the TMPMM struggles to achieve high-performance half-wave plate functionality at room temperature. When $S = 1/4$ and $\sigma_{VO_2} = 2 \times 10^5$ S/m, the cross-polarized component T^f_{xy} was not effectively suppressed upon the incidence of y-polarized wave (as shown by the orange curve in Figure S10(i)). As $T^f_{xy} = T^b_{yx}$, the TMPMM cannot be effectively functioned as isolator for x-polarized waves at higher temperatures. These results are consistent with the analysis in Figure S10(c) and (d). To quantitatively describe the dependence of the operating frequency on the scaling factor S, Figure S10(s) presents the peak frequency of T^f_{yx} as a function of S^{-1}, together with a linear fit. It can be observed that the operating frequency of the TMPMM scales linearly with S^{-1}, and its operating band can be effectively extended from 16.2 GHz to 7 THz, covering important communication bands such as Ku, K, Ka, V, W, and millimeter wave bands [33].

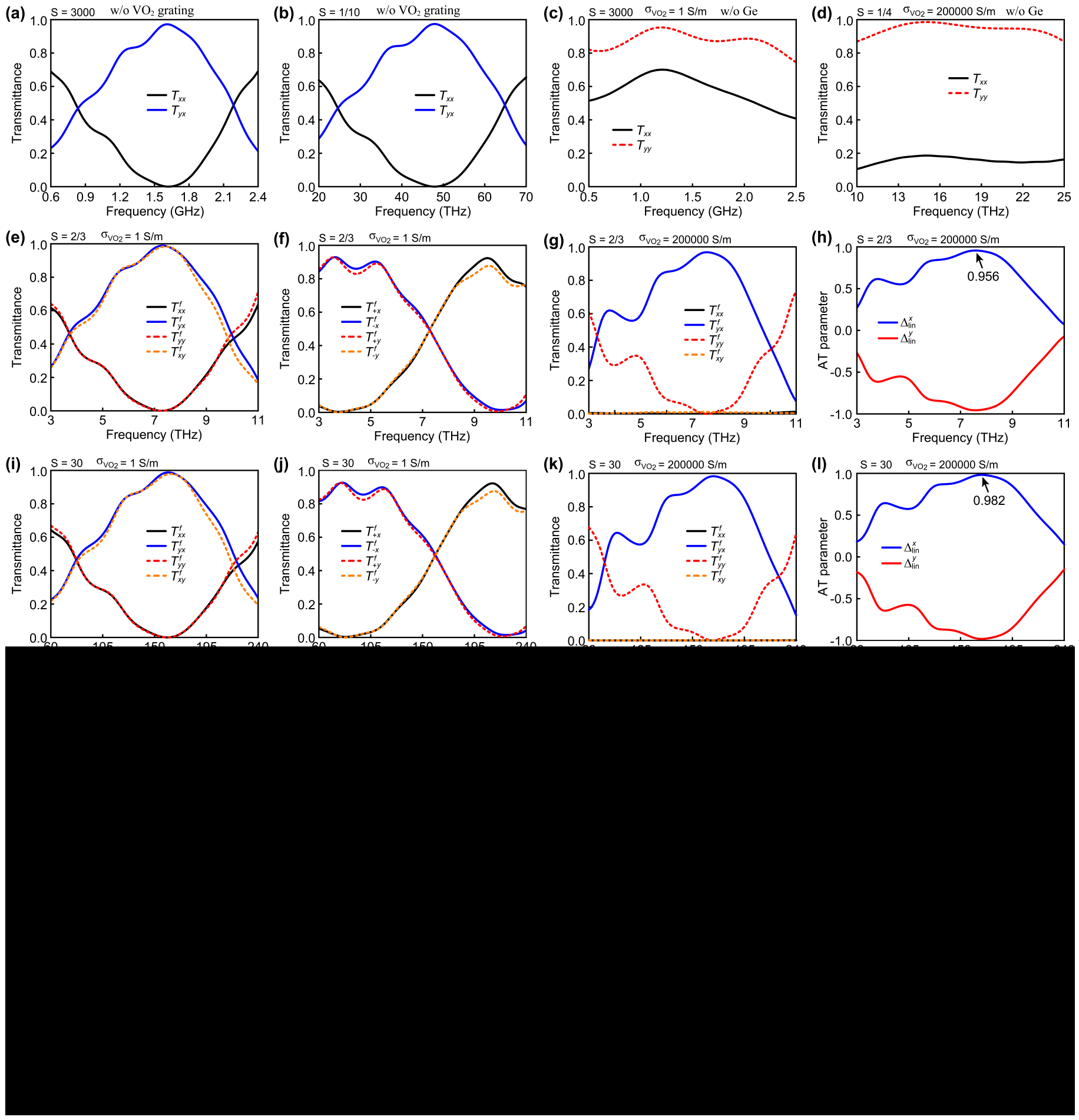

[redacted]

Figure S10. Co/cross polarization transmittances of x-polarized waves by metasurface structure without VO_2 grating at (a) $S = 3000$ and (b) $S = 1/10$. Co-polarization transmittances by VO_2 grating without Ge pillars at (a) $S = 3000$ and (b) $S = 1/4$. The corresponding performances of the TMPMM structure functioning as a half-wave plate, quarter-wave plate, and asymmetric transmission device at (e)-(h) $S = 2/3$, (i)-(l) $S = 30$, and (m)-(p) $S = 300$. Co/cross polarization transmittances of the TMPMM for different polarization waves at (q) $S = 3000$ and (r) $S = 1/4$. (s) Peak frequency of the cross-polarization transmittance T^f_{yx} as a function of S^{-1}.

References

[1] E. W. Wang, T. Phan, S.-J. Yu, S. Dhuey, J. A. Fan, *Proc. Natl. Acad. Sci. USA* **2022**, *119*, 12 e2122085119.

[2] W. Wang, J. Wang, Q. Wu, J. Lin, P. Jin, S. Liu, Z. Guo, K. Zhou, *Nanophotonics* **2026**, *15*, 2 e70012.

[3] T.-Y. Lin, S.-H. Huang, P.-C. Chen, Y.-C. Lin, C.-P. Lin, S.-Y. Chen, P. C. Wu, *Nano Lett.* **2025**, *25*, 26 10579.

[4] Y. Ren, B. Tang, *J. Lightwave Technol.* **2021**, *39*, 18 5864.

[5] Y. Wang, S. Li, H. Wang, L. Feng, B. Tan, Y. Tan, R. Su, J. Wu, C. Zhang, B. Jin, J. Chen, P. Wu, *Appl. Phys. Lett.* **2022**, *121*, 15 151701.

[6] J. Zhao, N. Li, Y. Cheng, *Chin. Opt. Lett.* **2023**, *21*, 11 113602.

[7] N. Yan, J. Zhang, F. Yang, Y. Luo, K. Ma, *IEEE Antennas and Wireless Propag. Lett.* **2025**, *24*, 11 4272.

[8] T. Q. H. Nguyen, T. M. Nguyen, *Adv. Theor. Simul.* **2025**, *8*, 2 2400817.

[9] J. Bai, Y. Yao, *ACS Nano* **2021**, *15*, 9 14263.

[10] Y. Zhao, R. Yang, Y. Wang, W. Zhang, J. Tian, *Opt. Express* **2022**, *30*, 15 27407.

[11] Q. Zhou, Q. Qiu, T. Wu, Y. Li, Z. Huang, *ACS Omega* **2023**, *8*, 50 48465.

[12] Y. Yan, Y. Liu, Q. Wang, J. Xu, *Opt. Laser Technol.* **2026**, *199* 115046.

[13] F. Lv, L. Wang, Z. Xiao, M. Chen, Z. Cui, Q. Xu, *Opt. Mater.* **2021**, *114* 110667.

[14] T. Lv, Y. Li, C. Qin, J. Qu, B. Lv, W. Li, Z. Zhu, Y. Li, C. Guan, J. Shi, *Opt. Express* **2022**, *30*, 4 5439.

[15] X. Tao, L. Qi, T. Fu, B. Wang, J. A. Uqaili, C. Lan, *Opt. Laser Technol.* **2022**, *150* 107932.

[16] F. Yin, Y. Lv, D. Xu, X. R. Jin, Y. Q. Zhang, *Opt. Express* **2023**, *31*, 17 27171.

[17] A. Arbabi, A. Faraon, *Sci. Rep.* **2017**, *7*, 1 43722.

[18] D. L. Markovich, A. Andryieuski, M. Zalkovskij, R. Malureanu, A. V. Lavrinenko, *Appl. Phys. B* **2013**, *112*, 2 143.

[19] Y. Gao, Y. Tian, Q. Du, Y. Wang, Q. Fu, Q. Bian, Z. Li, S. Feng, F. Ren, *Chinese Phys. B* **2023**, *32*, 7 074201.

[20] M. Jia, Z. Wang, H. Li, X. Wang, W. Luo, S. Sun, Y. Zhang, Q. He, L. Zhou, *Light: Sci. Appli.* **2019**, *8*, 1 16.

[21] C. C. Popescu, K. Aryana, P. Garud, K. P. Dao, S. Vitale, V. Liberman, H.-B. Bae, T.-W. Lee, M. Kang, K. A. Richardson, M. Julian, C. A. R. Ocampo, Y. Zhang, T. Gu, J. Hu, H. J. Kim, *Adv. Mater.* **2024**, *36*, 36 2400627.

[22] S. Wu, S. Zhu, K. T. Lai, L. Hou, V. Pusino, D. R. Cumming, *Laser Photonics Rev.* **2026**, *20*, 4 e01360.

[23] H. Jiang, J. Zhang, F. Yang, F. Chen, Y. Liu, H. Zhao, Y. Tang, Y. Zhang, M. Li, *Laser Photonics Rev.* **2026**, *20*, 10 e02428.

[24] D. Liu, D. Xia, T. Lv, J. Han, B. Yu, J. Shi, *Phys. Lett. A* **2026**, *575* 131397.

[25] Y. Ren, B. Tang, *J. Lightwave Technol.* **2021**, *39*, 18 5864.

[26] W. Liu, J. Y. Dai, Y. M. Zhang, M. K. Sun, L. Zhang, Y. Li, H. J. He, T. J. Cui, Q. Cheng, *Laser Photonics Rev.* **2024**, *18* 2400049.

[27] H. Cai, J. A. Dolan, G. S. D. Gordon, T. Chung, D. López, *ACS Photonics* **2021**, *8*, 9 2581.

[28] Y. Deng, Z. Cai, Y. Ding, S. I. Bozhevolnyi, F. Ding, *Nanophotonics* **2022**, *11*, 10 2219.

[29] Y. Li, I. Liberal, N. Engheta, *Sci. Adv.* **2019**, *5*, 10 eaav3764.

[30] A. Ahmed, M. R. Robel, W. S. T. Rowe, *IEEE Antennas and Wireless Propag. Lett.* **2020**, *19*, 4 641.

[31] W. Liu, L. Shi, J. Zi, C. T. Chan, *Nanophotonics* **2021**, *10*, 17 4297.

[32] F. Zhu, K. Yang, J. Hao, K. Fan, H. Ma, J. Wu, C. Zhang, X. Zhang, H. Wang, B. Jin, J. Chen, P. Wu, *Laser Photonics Rev.* **2025**, *19*, 8 2401732.

[33] J. A. Bruder, *IEEE Aerosp. Electron. Syst. Mag.* **2013**, *28*, 7 19.
